# Coconut rhinoceros beetle digestive symbiosis with potential plant cell wall degrading microbes

**DOI:** 10.1038/s41522-024-00505-9

**Published:** 2024-03-30

**Authors:** Chiao-Jung Han, Chih-Hsin Cheng, Ting-Feng Yeh, Yannick Pauchet, Matan Shelomi

**Affiliations:** 1 Department of Entomology, National Taiwan University, Taipei, Taiwan; 2 School of Forestry & Resource Conservation, National Taiwan University, Taipei, Taiwan; 3 Department of Insect Symbiosis, Max Planck Institute for Chemical Ecology, Jena, Germany

**Keywords:** Symbiosis, Microbial ecology

## Abstract

Coconut rhinoceros beetle (CRB, *Oryctes rhinoceros*) is an invasive palm pest whose larvae eat wood, yet lack the necessary digestive enzymes. This study confirmed endogenous CRB cellulase is inactive, suggesting microbial fermentation. The inner lining of the CRB hindgut has tree-like structures covered with a conspicuous biofilm. To identify possible symbionts, 16 S *rRNA* amplicon sequencing was used on individuals from across Taiwan. Several taxa of Clostridia, an anaerobic class including many cellulolytic bacteria, were highly abundant in most individuals from all locations. Whole metagenome sequencing further confirmed many lignocellulose degrading enzymes are derived from these taxa. Analyses of eggs, larvae, adults, and soil found these cellulolytic microbes are not transmitted vertically or transstadially. The core microbiomes of the larval CRB are likely acquired and enriched from the environment with each molt, and enable efficient digestion of wood.

## Introduction

Many herbivorous Coleoptera larvae feed on material consisting mainly of plant cell walls, suggesting the presence of plant-cell wall-degrading enzymes (PCDWEs)^1,2^. PCDWEs target pectin, lignin, cellulose, or hemicelluloses such as xylan and mannan^3,4^. PCWDEs include enzymes in several glycosidase hydrolase (GH) families, as well as some enzymes in carbohydrate esterase (CE), polysaccharide lyase (PL)^5^, and auxiliary activities (AA) families, and frequently have carbohydrate binding modules (CBM)^6^. These enzymes are produced by intestinal microbes or the insect itself, or both. Some endogenous insect genes may have been acquired from microbes through horizontal gene transfer^7,8^, while some may have been present in the common ancestor of insects^9^. Insects that produce endogenous PCWDEs can thus either digest plant matter independently of microbes, or with them through synergistic production of complementary enzymes.

Scarabs (Scarabaeidae) comprise more than 30,000 species worldwide^10,11^. More than 70% of them are phytophagous^10,11^. Recent research by McKenna et al^5^. suggested that the Scarabaeoidea generally have genes for GH1 (cellobiase) and GH9 (cellulase), but lack most other PCWDEs in their genomes compared to other herbivorous beetle superfamilies like Chrysomeloidea and Curculionoidea. This strongly implies that the Scarabaeoidea need microbial assistance in digestion^5^, especially those that feed on wood. A study of the flower-chafer (*Protaetia brevitarsis*, Scarabaeidae) larval holobiont revealed that the beetle selectively enriches lignocellulose degrading microbes, particularly species of Firmicutes (syn. Bacillota) and Bacteroidetes (syn. Bacteroidota), which do most of the lignocellulose degradation instead of the host’s endogenous enzymes^6^. Microbiome studies of Japanese beetles (*Popillia japonica*, Scarabaeidae) and dung beetles (*Copris incertus*, Scarabaeidae) further suggest microbial PCWDEs facilitate digestion in scarab larvae^12,13^.

Certain microbes may be irreplaceably significant, particularly in Scarabaeidae with difficult-to-digest diets^14^. For example, *P. japonica* has a stable microbial community across all developmental stages, including clades well-known for PCWDE production such as Ruminococcaceae, Christensenellaceae, and Lachnospiraceae, which were mostly not present in the environment. This suggests the gut microbiome is important for maintaining the normal physiological state of the host, and is maintained with direct transmission of the microbes from parent to offspring^12^. Such symbiotic relationships are often associated with specialized intestinal morphology, such as bacteriomes or mycetomes to store intracellular symbionts as in *Costelytra zealandica* (Scarabaeidae)^15,16^, or extracellular structures like fermentation chambers, crypts, and mycangia as in *Cephalodesmius* spp. (Scarabaeidae) dung beetles^17^.

Coconut rhinoceros beetles (CRB, *Oryctes rhinoceros*) are infamous pests on Arecaceae plants, especially oil palms, areca trees, and coconut trees^18,19^. They originated from countries in Southeast Asia, with Taiwan considered one of their original habitats. CRBs rapidly spread into the Pacific, reaching numerous CRB-free territories over the last century, including recent infestations in the US and Mexico. The United States Department of Agriculture (USDA) Animal and Plant Health Inspection Service (APHIS) considers CRBs invasive and alien pests^18,20–22^. Adults target the growing points of young trees, and a single individual is capable of damaging multiple trees. They preferentially oviposit on decaying fibers such as stalks, rotten wood, and herbivore manure, likely guided by olfactory signaling^23^. Larvae are mainly stalk borers, predominantly feeding on the decaying plant tissues. As a result, trees infested by CRB become vulnerable to collapse, or fungal infections through wounds^24^. As scarab beetles, they are expected to depend on microbes for much of their enzyme production. A transcriptomics study of the CRB gut found only one GH9 cellulase gene (Accession number: MN047310) with surprisingly low expression^25^, suggesting the enzyme may be inactive and further implying microbial PCWDEs are necessary for digestion. A culturing-based microbiome analysis of CRB found several cellulolytic and hemicellulolytic strains of *Bacillus* spp. and *Citrobacter koseri*^4^, however these are ubiquitous microbes found in many other environments. Another study using both culturing and metagenomics found either *Citrobacter koseri* or *Paracoccus* sp. in every individual larva^26^, however whether these microbes assist in digestion remained unclear.

This study aimed to first confirm PCWDE activity in the gut, then identify what activities are attributable to the endogenous CRB cellulase and what to isolated gut microbes. The diversity and composition of the larval microbiome was profiled using 16 S *rRNA* gene metabarcoding and whole genome sequencing used to identify the responsible taxa expressing different PCWDEs. To find evidence of a long-term symbiosis, gut anatomy was examined in detail and the microbiome in different stages of development surveyed. This study unveils the digestive process for lignocellulosic matter in CRB’s holobiont, and the microbes or genes identified could have possible industrial applications in the biofuel production^27^.

## Results

### CRB degradation of cellulose and hemicellulose

To examine whether CRB can depolymerize their diets, first the degradation of cellulose and hemicelluloses was confirmed with wet chemistry methods and nuclear magnetic resonance spectroscopy (NMR) by comparing the content changes and the chemical shift between their diet (wood) and feces. To complement the wet chemistry and NMR results, PCWDE bioassays were performed on the digestome of CRBs from different stages of development, CRB endogenous cellulase expressed in vivo, and isolated gut microbes.

The lignin, neutral sugars, and uronic acid contents of the diet and feces are given in Table 1. The feces had significantly reduced rhamnose, arabinose, xylose, galactose and uronic acid contents (*p* < 0.05). These neutral sugars were from the hemicelluloses of the biomass. Glucose, which is predominantly from cellulose, was also significantly reduced. These results indicated that the cellulose and hemicelluloses of the diet were digested by CRB. By contrast, while total lignin content (the summation of acid-insoluble and acid-soluble lignins) of the feces was not different from the diet by the *t*-test, the acid-soluble lignin content of the feces was higher than the diet, indicating that the polymer lignin was digested by the beetles into small fragments soluble in a weak sulfuric acid solution.

**Table 1:** Lignin, neutral sugar, and uronic acid contents of CRB’s diet and feces

Content (%)	Cocopeat	Frass
Acid-Insoluble Lignin (AIL)	50.33 ± 1.50	51.12 ± 0.86
Acid-Soluble Lignin (ASL)^a^	0.91 ± 0.04	2.00 ± 0.10
Rhamnose^a^	0.45 ± 0.04	0.40 ± 0.04
Arabinose^a^	4.68 ± 0.28	2.40 ± 0.07
Xylose^a^	8.25 ± 0.44	5.88 ± 0.17
Mannose	1.23 ± 0.07	1.19 ± 0.02
Glucose^a^	20.54 ± 0.46	15.71 ± 0.53
Galactose^a^	1.62 ± 0.09	1.26 ± 0.12
Uronic Acid^a^	2.02 ± 0.03	1.70 ± 0.05

Ratio is based on extractive-free oven-dried sample weight. Data of AIL are mean ± standard deviation (SD) (*n* = 3). Others are mean ± SD (*n* = 9). Total lignin can be calculated from (AIL + ASL).

^a^Significant difference between the cocopeat and feces at *p* < 0.05 with two-tailed *t*-test.

**Fig. 1:**
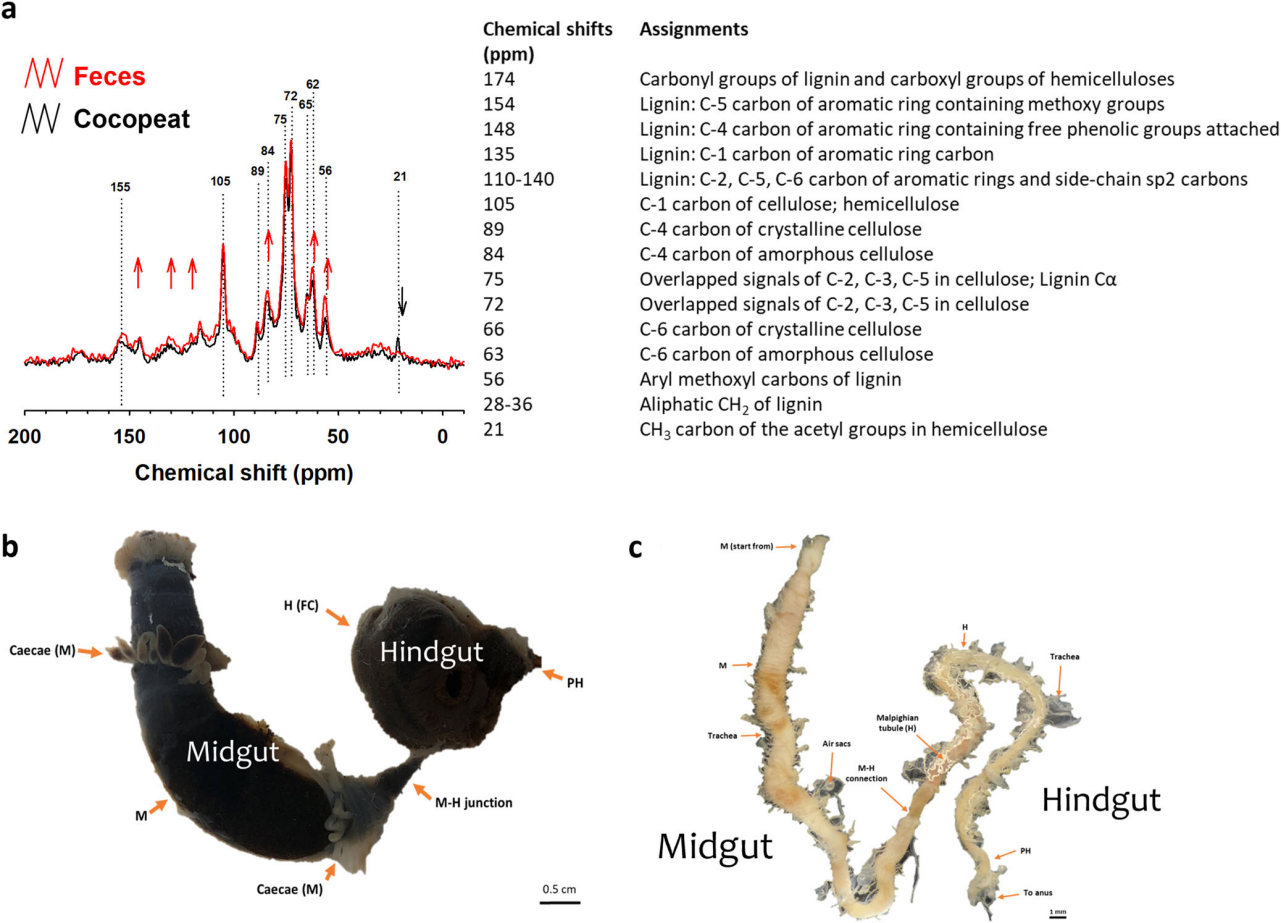
The digestive activity in the gut of CRB. **a** The chemical shift (ppm) from the solid-state ^13^C nuclear magnetic resonance spectroscopy (NMR) of cocopeat and feces. **b** The digestive tract of the third instar larva. The scale bar represents 0.5 cm. **c** The digestive tract of the adult. The scale bar represents 1 mm. The abbreviations in (**b**) and (**c**) are defined as follows: M midgut, H hindgut, FC fermentation chamber, M-H junction midgut-hindgut junction, PH posterior hindgut, M-H connection midgut-hindgut connection.

The solid-state ^13^C NMR data covered a total range from 290.7831 ppm to −123.9502 ppm. Peaks in chemical changes in wood materials with chemical shifts from 200 ppm to −10 ppm are depicted in Fig. 1a. The comparison showed significant differences between undigested and digested wood. The signal assigned to the methyl carbon of the acetyl groups in hemicellulose disappeared in the feces samples, and the signal for the C-1 carbon of hemicellulose (105 ppm) was slightly reduced, suggesting hemicellulose digestion occurred. The signals assigned to lignin increased (154 ppm, 148 ppm, 135 ppm, 75 ppm, 56 ppm), suggesting lignin compounds were preserved. Changes in cellulose (105 ppm, 89 ppm, 84 ppm, 75 ppm, 72 ppm, 66 ppm, 63 ppm) were not evident, but the ratios of amorphous (84 ppm, 63 ppm) to crystalline (89 ppm, 66 ppm) C increased, showing the formation of more amorphous compounds during digestion and suggesting degradation of cellulose and hemicellulose (Fig. 1a).

PCWDE activity of digestive tract contents from CRB late instar larvae (Fig. 1b), pupae^28^, and adults (Fig. 1c) was examined by using plate assays with four different substrates: carboxymethylcellulose (CMC), xylan, xyloglucan, and glucomannan (Supplementary Fig. 1). The larval midgut contents, the hindgut wall, and the hindgut contents showed cellulase activity against CMC in a pH range of 6–10 and the highest performance at pH 8. No apparent xylanolytic activity against xylan was observed in any samples. Xyloglucan degradation was observed for larval midgut contents at pH 9, hindgut wall at pH 8, and hindgut contents at pH 8–9. Glucomannan degradation was observed for larval hindgut contents at pH 8. Overall, the larval hindgut contents had the most versatile digestive ability and enzyme activity was highest at pH 8–9. Neither the pupal nor the adult digestive tracts showed activity against any of the substrates.

**Fig. 2:**
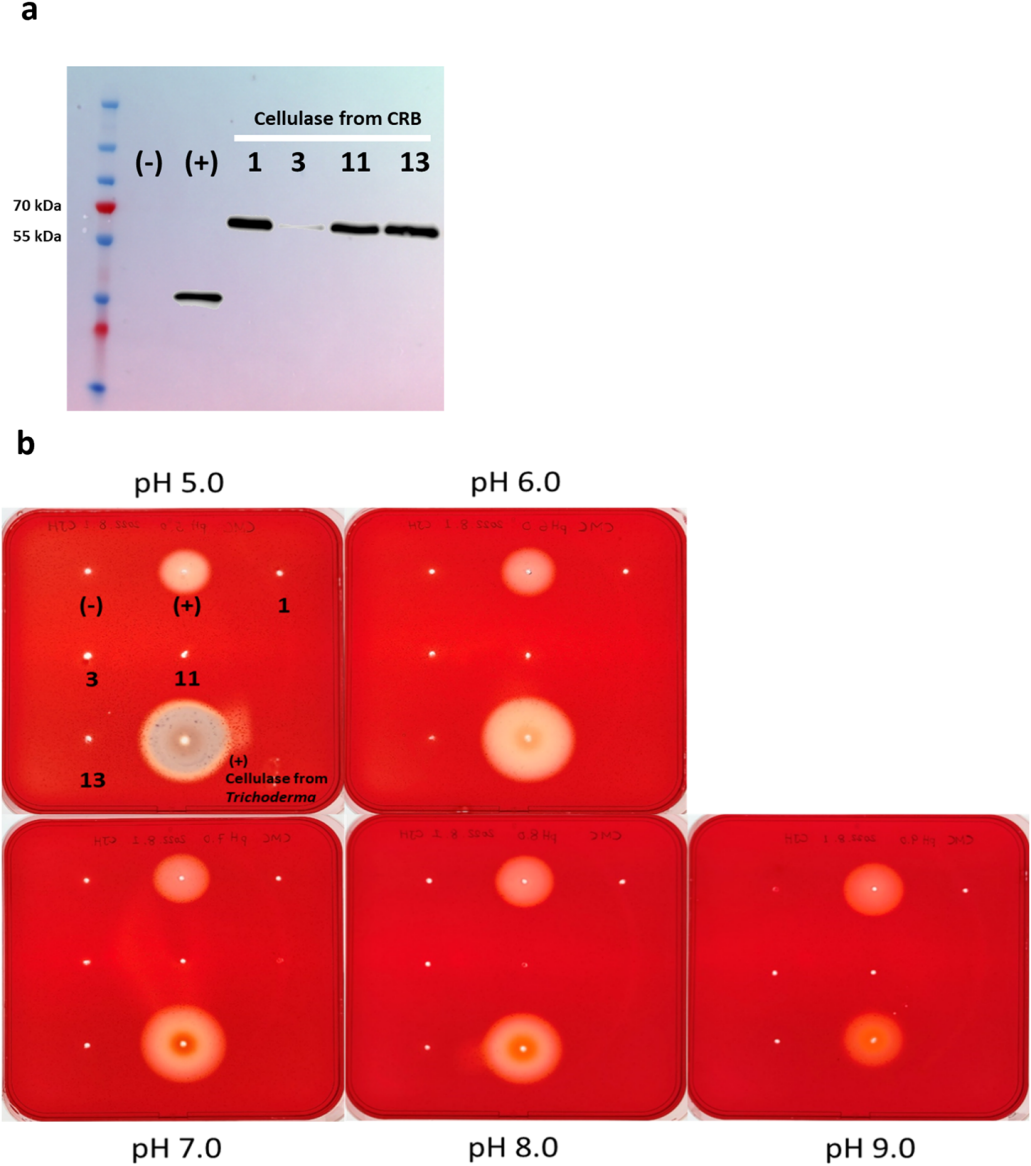
Plate assays for endogenous cellulase activity. **a** Western blot of the cellulase from CRB expressed by *Sf9* cells. Positive control (+) is GH45 cellulase from a leaf beetle (*Cassida rubiginosa*) expressed by *Sf9* cells. Code 1, 3, 11, 13, are the transfectants of CRB’s cellulase with protein expression in the size of approximately 65 kDa. However, the protein expression level from the transfectant 3 is nearly unobservable. **b** Plate assays for endogenous cellulase activity ranging from pH 5.0–9.0 by Congo red staining. Codes 1, 3, 11, and 13 are cellulases of CRB expressed from transfectants, none of which showed CMC-ase activity under pH 5.0–9.0. The positive controls were cellulase from *Trichoderma reesei*, and cellulase expressed from *Cassida rubiginosa*.

**Fig. 3:**
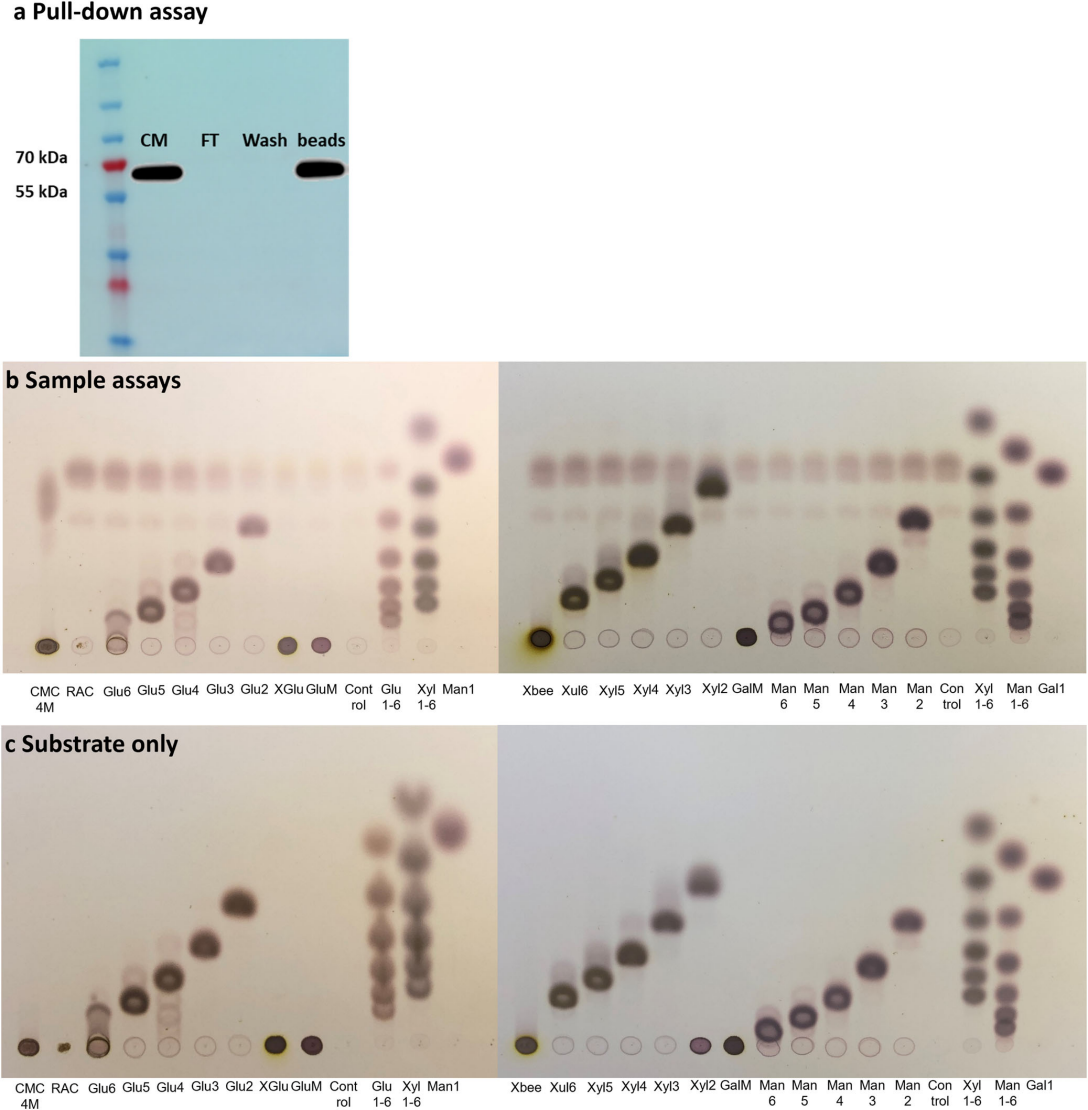
Thin layer chromatography (TLC) assays with different substrates to test GH9 activity. **a** Western blot of the enzyme after pull-down assays for thin layer chromatography (TLC) assays. Beads: the beads that attach the cellulase; CM: the cells with culture media; FT: the first flow-through during the purification process; Wash: the flow-through during the wash step in the purification process. The transfectant of CRB’s cellulase with protein expression in the size of approximately 65 kDa from the CM can be seen, which is also consistent with the protein size on the beads, suggesting a successful purification process. During the purification processes, no cellulase was detected in the FT and Wash, indicating the recovery of the protein was efficiently processed. **b**, **c** TLC assays with different substrates to test GH9 activity. **b** Sample assays. **c** Control assays without sample enzymes. No visible degradation of the substrates can be seen, suggesting the cellulase is inactive. Abbreviations as defined in the legend of Supplementary Table 1.

The endogenous CRB GH9 cellulase was expressed in an *Sf9* cell line (Fig. 2a) and the activity tested by plate assays. No observable CMC-ase activity was observed (Fig. 2b). After confirming the successful binding of the recombinant GH9 to anti-V5 beads with a Western blot (Fig. 3a), thin layer chromatography (TLC) assays with different substrates found no visible degradation of any of the substrates (Fig. 3b, c).

The microbes isolated from CRB that had tested positive for PCWDE activity assays are listed in Supplementary Table 1. Firmicutes, especially *Bacillus*, generally had PCWDE activity. In addition, many yeasts in the Ascomycota phylum (*Candida*, *Trichosporon*, and *Pascua)* also had PCWDE activity.

### Scanning electron microscopy revealed a larval hindgut biofilm

**Fig. 4:**
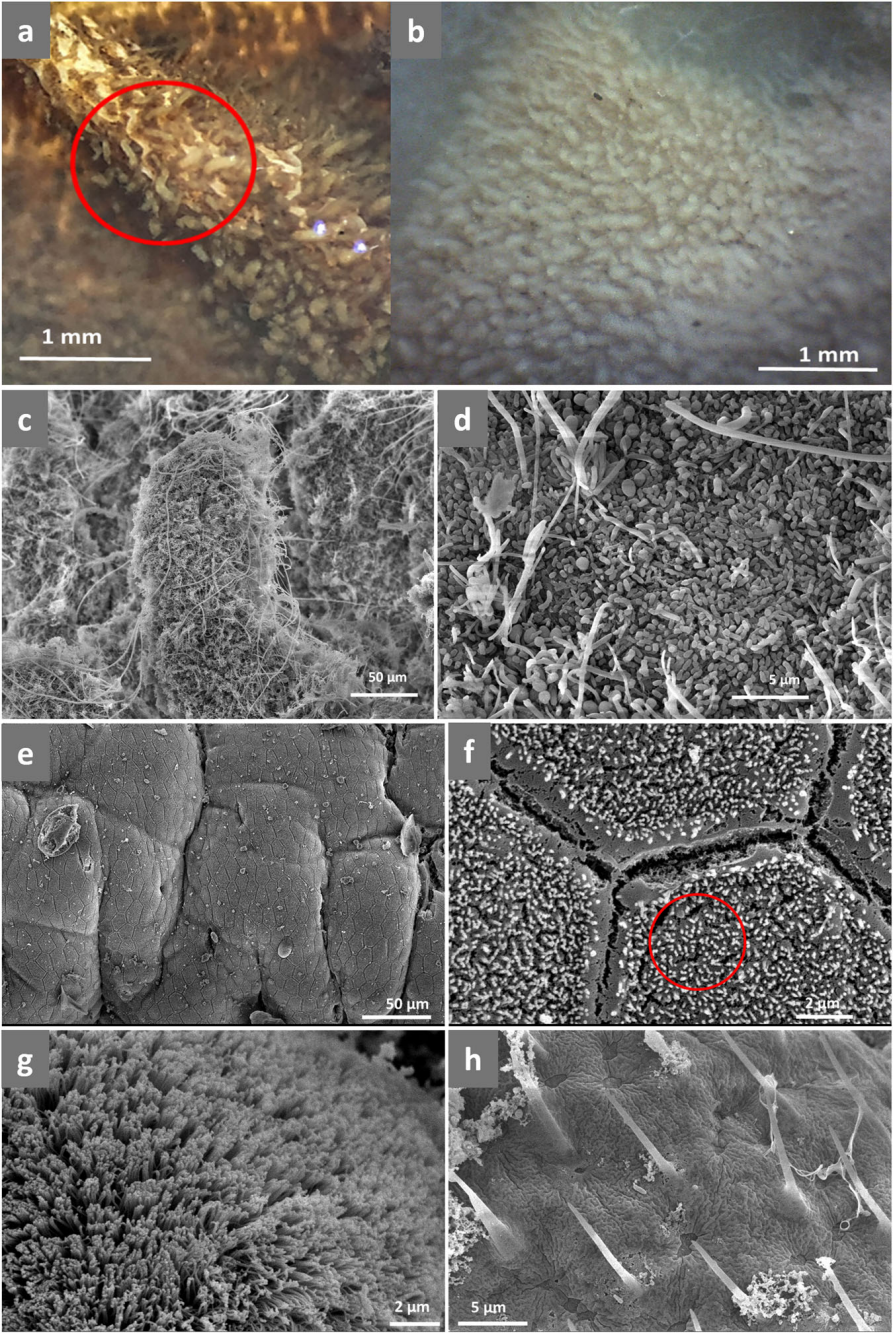
The symbiotic structures of CRB. **a** Stereomicrograph of the tree-like structures on a larval CRB hindgut. The scale bar represents 1 mm. **b** Stereomicrograph of the tree-like structures on a larval CRB hindgut. The scale bar represents 1 mm. **c** Scanning electron microscope (SEM) image of the tree-like structures showing a conspicuous biofilm. The scale bar represents 50 μm. **d** The bacterial filaments and cells comprising the biofilms. The scale bar represents 5 μm. **e** SEM image of the larval midgut showing no tree-like structures. The scale bar represents 50 μm. **f** Microvilli on the larval midgut inner lining. The scale bar represents 2 μm. **g** Microvilli on the adult midgut inner lining. The scale bar represents 2 μm. **h** “Naked tree-like structures” lacking biofilms on the adult hindgut inner lining. The scale bar represents 5 μm.

Tree-like structures were observed in the larval hindgut by stereomicroscope (Fig. 4a, b), and hypothesized to have symbiotic functions. Detailed scanning electron micrographs of the structures revealed that they are covered in a conspicuous biofilm (Fig. 4c) composed of bacterial filaments and cells (Fig. 4d). Such biofilm structures could not be seen in the larval midgut (Fig. 4e, f) nor in the adult midgut or hindgut (Fig. 4g, h).

### General description of the larval microbiome and the interaction of core microbes

**Fig. 5:**
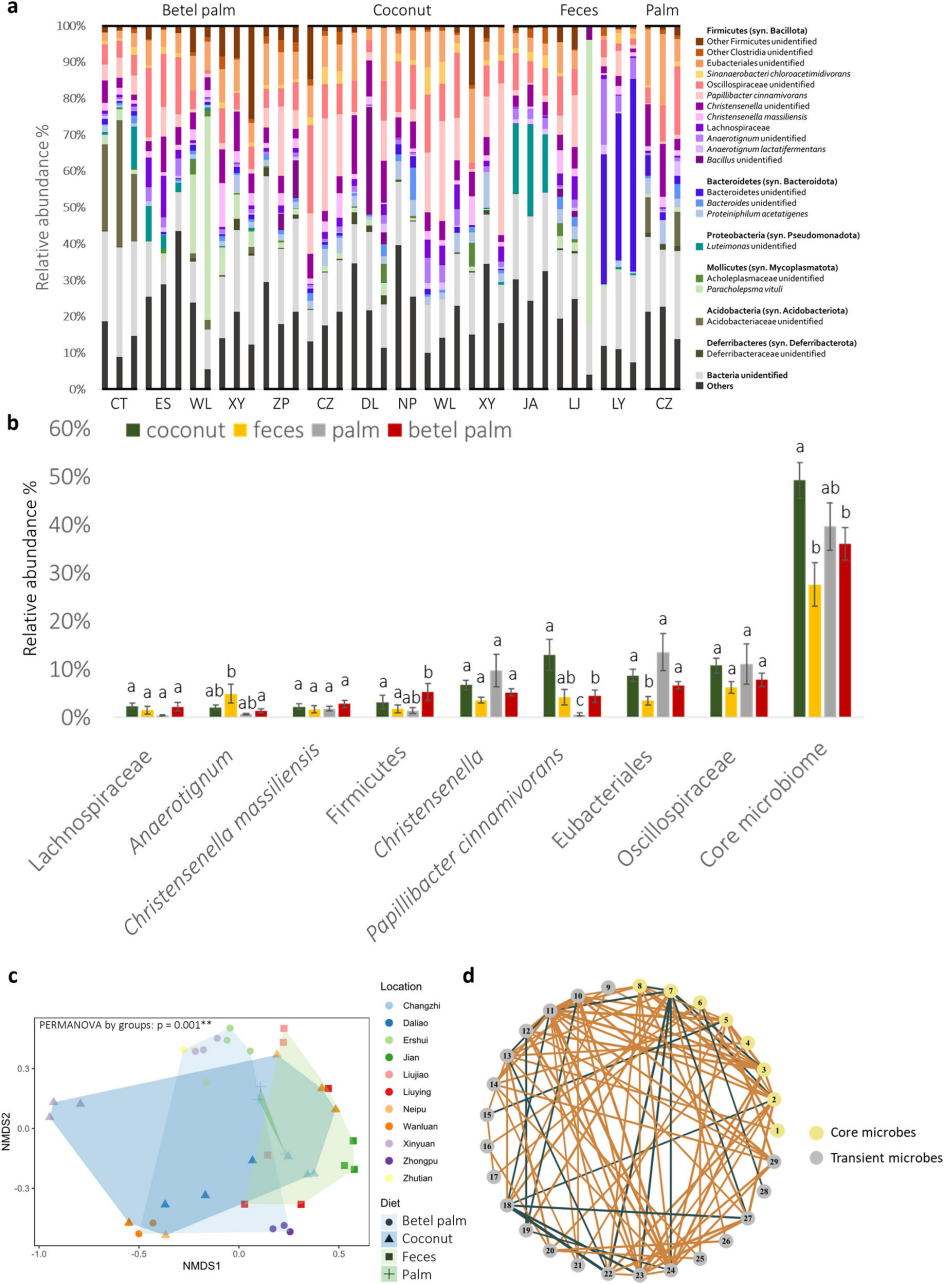
Analyses of microbiome and the core microbes of CRB. **a** The relative abundance by taxonomic composition among the top 20 most abundant bacterial ASVs from the late larval microbiome. ASVs with a frequency of occurrence lower than 50% are not shown in the figure. Abbreviations of host plant on the top and locations at the bottom as defined in the Supplementary Table 1. Each bar represents a larva’s microbiome. **b** The relative abundances of the eight core microbes (present in 90% or more individuals) among all sampled individuals separated by diet. Kruskal–Wallis test and pairwise Wilcox test are used for the hypothesis tests, with a significance level of 0.05. The error bar represents for the standard error (SE) (**c**) Non metric multidimensional scaling (NMDS) plot by Bray–Curtis distance (Stress = 0.12). Test of significance was conducted by using PERMANOVA. Significant differences were found among diet and location group (*p* = 0.001) and between all pairs of locations and diets, except for coconut and palm whose microbiome communities did not differ significantly (*p* > 0.05). **d** Network correlational analysis of the core microbes and transient microbes. Orange lines indicate positive correlations like mutualism or commensalism between the two microbes, whereas green lines indicate negative correlations like competition, antagonism, or predation. The thicker the line, the stronger the strength of the interaction. The yellow nodes are core microbes, gray nodes are transient microbes. The corresponding microbes for each number are: 1-*Papillibacter cinnamivorans*; 2-*Christensenella massiliensis*; 3-Eubacteriales; 4-Oscillospiraceae; 5-*Christensenella* sp.; 6-Firmicutes; 7-Lachnospiraceae; 8-*Anaerotignum* sp.; 9-*Proteiniphilum acetatigenes*; 10-*Anaerotignum lactatifermentans*; 11-Clostridia; 12-*Sinanaerobacter chloroacetimidivorans*; 13-*Bacteroides*; 14-*Desulfovibrio cuneatus*; 15-*Paludibacter propionicigenes* WB4; 16-Bacteroidetes; 17-*Paracholepsma vituli*; 18*-Sporobacter termitidis*; 19-Deferribacteraceae; 20-*Ruminiclostridium*; 21-*Propionispora vibrioides*; 22-Peptococcaceae; 23-Dysgonomonadaceae; 24-*Pseudoflavonifractor phocaeensis*; 25-*Oxalobacter vibrioformis*; 26-*Phocea massiliensis*; 27-Bacteroidia; 28-*Anaerotruncus rubiinfantis*; 29-Alphaproteobacteria.

To screen the core microbes from CRB, 16 S full-length metabarcodes were sequenced from the hindgut contents of 40 third instar larvae with four different diets and from 11 different locations in Taiwan, totaling 591,034 reads with 131,649 high-quality and filtered sequences producing 2178 amplicon sequence variants (ASVs), with a median of 3291 reads per sample and 168 ASVs per sample. The details of samples and the number of PacBio reads were displayed in Supplementary Table 2. A total of 16 phyla, 27 classes, 40 orders, 60 families, 85 genera, and 73 species were classified. Bacillota (syn. Firmicutes) (50 ± 2.9% of ASVs per individual), Bacteroidota (syn. Bacteroidetes) (10 ± 1.7%), and Pseudomonadota (syn. Proteobacteria) (7 ± 1.3%) were the three most abundant phyla. Among these phyla, species in the Bacillota class Clostridia were the most common clades from almost every sample (17–80% of total ASVs, 43 ± 2.8% per individual), followed by Mollicutes (6 ± 2% per individual) and Bacteroidia (6 ± 0.7% per individual, Fig. 5a). If a specific taxon was present in at least 90% of all sampled individuals, then it was defined as a member of the core microbial community. This core consisted of an unidentifiable member of Oscillospiraceae (8.6 ± 0.8% of ASVs per individual), an unidentifiable Eubacteriales (7.1 ± 0.7%), *Papillibacter cinnamivorans* (7.0 ± 1%), *Christensenella* sp. (5.7 ± 0.5%), *Christensenella massiliensis* (2.3 ± 0.3%), *Anaerotignum* sp. (2.2 ± 0.4%), and an unidentifiable Lachnospiraceae (2.0 ± 0.4%), all of which are in the class Clostridia, as well as an unidentifiable Bacillota/Firmicutes (3.5 ± 0.8%). Samples from most locations had these eight clades in the hindgut, except for an outlier individual: a larva found in cow feces that lacked most of the commonly occurring clades and whose gut was dominated by the Mollicute species *Paracholepsma vituli*. Some of these microbes correlated with diet (Fig. 5b). Non-multidimensional scaling plots (NMDS) showed that the microbial communities varied significantly with diets and location (Fig. 5c) (*p* = 0.001), although the microbiomes for larvae collected from coconut and *Phoenix* palms were not significantly different from each other (*p* > 0.05).

To see the compositional differences between hindgut contents and tissues, the comparison of microbiomes between 3rd instar larval hindgut contents and tissues is displayed in Supplementary Fig. 2, compared by using five pairs of hindgut contents and tissues from CTa, CZp, JAf, LYf, WLc samples. Code numbers can be referred to in Supplementary Table 3, and the details of samples and the number of PacBio reads were displayed in Supplementary Table 2. A total of 16 phyla, 24 classes, 97 orders, 90 families, 72 genera, and 57 species were classified. Bacillota (syn. Firmicutes), Bacteroidota (syn. Bacteroidetes), and Pseudomonadota (syn. Proteobacteria) were the three most abundant phyla (Supplementary Fig. 2a). Some ASVs were shared among hindgut contents and tissues: an unidentifiable member of Bacillota (syn. Firmicutes), an unidentifiable member of Clostridia (Bacillota), an unidentifiable member of Eubacteriales (Bacillota), an unidentifiable member of Oscillospiraceae (Bacillota), *Christensenella massiliensi*s, an unidentifiable member of *Christensenella*, and *Desulfovibrio cuneatus*. ASVs identified as *Papillibacter cinnamivorans*, *Sinanaerobacter chloroacetimidivorans*, an unidentifiable member of Lachnospiraceae, and an unidentifiable member of *Anaerotignum* were shared among all hindgut content samples. ASVs identified as *Ruthenibacterium lactatiformans*, *Sporobacter termitidis*, an unidentifiable member of Bacteroidia, *Paludibacter propionicigenes* WB4, *Proteiniphilum acetatigenes*, an unidentifiable member of *Desulfovibrio*, and an unidentifiable member of Deferribacteraceae were shared among all hindgut tissue samples (Supplementary Fig. 2a). Pairwise PERMANOVA of hindgut contents and tissues found significant differences (*p* < 0.01) (Supplementary Fig. 2b). All microbiomes were analyzed by PICRUSt2 to identify potential PCWDE profiles. Functional profile structures were visualized by NMDS with PERMANOVA, showing no significant differences between hindgut contents and tissues (*p* > 0.05) (Supplementary Fig. 2c). The overall potential productions of ligninase, xylanase, cellulase, hemicellulase, pectinase, and overall PCWDE profiles did not differ between hindgut contents and tissues (*p* > 0.1) (Supplementary Fig. 2d).

Network correlational analysis of the core and transient microbes revealed some ASVs interacted positively with each other, indicating mutualistic or commensalistic relationships, while others were negatively correlated, indicating competitive, antagonistic (amensalistic), or predatory relationships (Fig. 5d). Among the core microbes, the unidentified Lachnospiraceae had mostly negative interactions with other microbes, both core and transient. Most of the microbes had positive or neutral relationships with each other.

### The microbiome dynamics in different stages of development

**Fig. 6:**
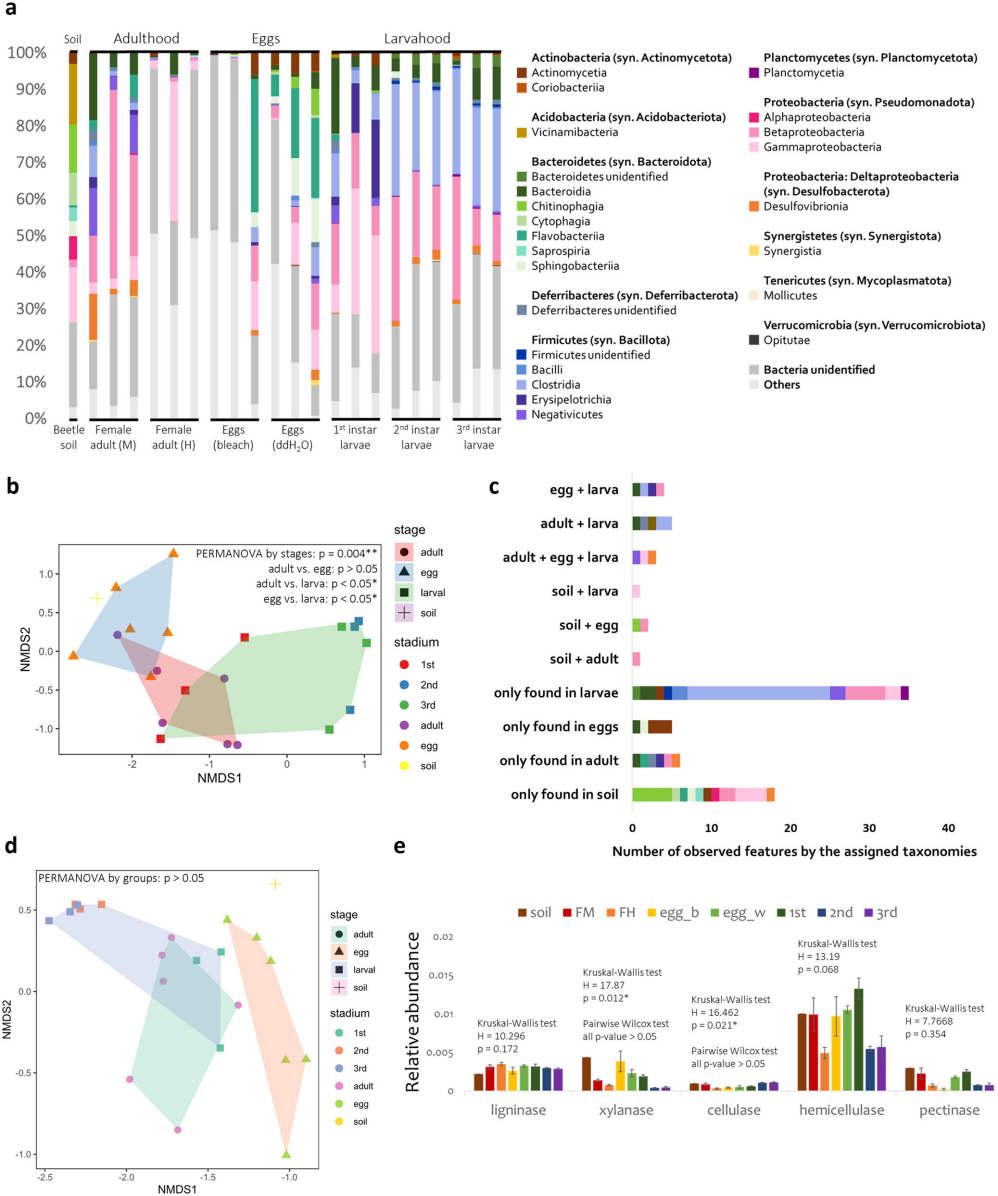
Analyses of microbiome in different stages of development and the predicted functional profiles. **a** The class level relative abundance of the microbiomes of different stages of CRB and the soil where they were reared. Each bar for female adults represents a single individual. Each bar for eggs represents three eggs from the same mother (female adult). Each bar for larvae, regardless of the stage, represents three individuals. The abbreviations of tissues at the bottom refer to the following: Female adult (M) for female adult midgut and Female adult (H) for female adult hindgut. **b** NMDS plot of microbiome by Bray–Curtis distance (stress = 0.082). Tests of significance were conducted by using PERMANOVA by different stage groups, *p* = 0.004. Pairwise PERMANOVA of adult and egg found no significant differences (*p* > 0.05, whereas significant distances were found between adult and larva and between egg and larva (*p* < 0.05). The abbreviations of stadium on the right refer to the following: 1st for first instar larva, 2nd for second instar larva, and 3rd for third instar larva. **c** The shared features of microbes between different stages of development. The color representations are indicated in (**a**). **d** The NMDS plot of the predicted functional profile of the microbiome by Bray–Curtis distance (stress = 0.012). Test of significance was conducted by using PERMANOVA. No significant differences were found between groups. The abbreviations of stadium on the right refer to the following: 1st for first instar larva, 2nd for second instar larva, and 3rd for third instar larva. **e** The relative abundance of the five PCWDE groups predicted according to the functional profile analysis. Test of significance was conducted by using Kruskal–Wallis test and pairwise Wilcox test by stadium. Kruskal–Wallis test of xylanase and cellulase showed significant differences between stadia (*p* < 0.05), but pairwise Wilcox tests found no significant differences (*p* > 0.05). The error bar represents for the standard error (SE). The abbreviations of tissues on the top refer to the following: soil for the soil utilized as the feeding substrate for these beetles, FM for female adult midgut, FH for female adult hindgut, egg_b for bleach treated eggs, egg_w for ddH_2_O rinsed eggs, 1st for the 1st instar larval whole gut, 2nd for the 2nd instar larval hindgut, and 3rd for the 3rd instar larval hindgut.

To check if microbial communities are transmitted interstadially from larvae to adults and vertically from adults to offspring, the 16 S full-length metabarcodes from egg tissues, the hindgut tissue from larvae of different stages of development, midgut and hindgut tissues of female adults, and the soil in which eggs were laid were sequenced. The resulting 532,402 reads, with 220,095 high-quality and filtered sequences produced 1078 ASVs with a median of 10,024.5 reads per sample and 75 ASVs per sample. The details of samples and the number of PacBio reads were displayed in Supplementary Table 2. A total of 11 phyla, 22 classes, 34 orders, 57 families, 121 genera, and 78 species were classified (Fig. 6a). NMDS of the microbiomes showed that the microbiome communities varied significantly between eggs, larvae, and adults (Fig. 6b). Pairwise PERMANOVA of adults and eggs found no significant differences between these two stages (*p* > 0.05) but found significant differences between adults and larvae and between eggs and larvae (*p* < 0.05).

Only three ASVs were shared among all developmental stages and were not detected from the soil: an unidentifiable Sporomusaceae (Bacillota, Negativicutes), an unidentifiable Enterobacteriaceae (Pseudomonadota: Gammaproteobacteria), and *Desulfovibrio cuneatus* (Desulfobacterota: Desulfovibrionia). ASVs identified as *Bacteroides* sp. (Bacteroidota: Bacteroidia), *Sphingobacterium* sp. (Bacteroidota: Sphingobacteriia), *Leucobacter* sp., *Microbacterium* sp., and *Xylanimonas ulmi* (Actinomycetota syn. Actinobacteria: Actinomycetia) were only found in the eggs (Fig. 6c). The eggs and adult midgut and hindgut had scarce microbes, with relatively lower microbial diversity and monotonous profiles compared to the larval microbiomes (Fig. 6a). Many ASVs were only found in larvae, followed by soil.

All microbiomes were analyzed by PICRUSt2 to identify potential PCWDE genes. Functional profile structures were visualized by NMDS with PERMANOVA (Fig. 6d), showing no significant differences between different stages of development. The overall potential productions of ligninase, xylanase, cellulase, hemicellulase, and pectinase did not differ by developmental stage (Fig. 6e).

### Lignocellulase-related enzyme modules in the CRB holobiont

To characterize PCWDEs, the prokaryotic metagenome and previous transcriptome data of the digestive tracts were mined for lignocellulose degradation-related modules (LDMs) including ligninases, cellulases, and hemicellulases, plus pectinases. A total of 705,518 contigs larger than 1 kilobase pair (Kbp) were produced from the metagenome reads, with an average N50 of 3.108 Kbp, a maximum contig size of 540.949 Kbp, and an average L50 of 122,017 contigs. In total, 2899 LDMs and 416 pectinases were found in the CRB holobiont, including 28 LDMs and 3 pectinases from the host alone, and 83 LDMs and 21 pectinases from the microbiome alone (Table 2). These modules consist of 4 families of auxiliary activity enzymes (AA), 24 carbohydrate binding modules (CBM), 12 families of carbohydrate esterases (CE), 67 families of glycoside hydrolases (GH), and 5 families of polysaccharide lyases (PL). The modules AA2, GH27, GH29, GH47, GH152, CBM1, CBM39, and CBM47 were only identified in the host; whereas dozens of different modules were only identified in the microbiome. As before, the only true cellulase from the CRB transcriptome was the one inactive GH9, although two putative, endogenous, GH3 beta-1,4-glucosidase transcripts were found. All other cellulases were microbial in origin. CE4, GH3, GH94, and GH2 enzymes were particularly common, and predominantly produced by microbes.

**Table 2:** CAZyme distribution from prokaryotic metagenome and CRB’s transcriptome

Type of enzymes	CAZyme modules	Microbiome (hindgut)		Host transcriptome			number of genes
		16 S Microbiome	Prokaryotic Metagenome	Midgut-only	Hindgut-only	Both	
Ligninases	AA1		2	2		3	7
	AA2	predicted				1	1
	AA3		9	1	1	3	14
	AA4		20			1	21
	CE15		12				12
Cellulases	GH6	predicted	6				6
	GH9	predicted	17	1			18
	GH55		8				8
	GH81		2				2
	GH94		125				125
	GH128		3				3
	GH144	predicted	19				19
Cellulases & hemicellulases	GH1	predicted	38				38
	GH3	predicted	222	2			224
	GH4	predicted	95				95
	GH5	predicted	75				75
	GH8	predicted	15				15
	GH16		36				36
	GH26	predicted	26				26
	GH30	predicted	26				26
	GH31	predicted	61				61
	GH39	predicted	22				22
	GH51	predicted	59				59
	GH74	predicted	2				2
	GH116	predicted	15				15
Hemicellulases	CE1		87	2		1	90
	CE2		2				2
	CE3		8				8
	CE4		477			1	478
	CE6		2				2
	CE7		14				14
	CE17		5				5
	CE20		43				43
	GH2	predicted	206		1	1	208
	GH10	predicted	87				87
	GH11	predicted	14				14
	GH27				2	1	3
	GH29				1	1	2
	GH35	predicted	8		1	1	10
	GH36		38				38
	GH38		68		3	3	74
	GH42	predicted	23				23
	GH43	predicted	155			1	156
	GH47	predicted		2	2	6	10
	GH53		16				16
	GH57		15				15
	GH59	predicted	2				2
	GH67	predicted	15				15
	GH76		18			1	19
	GH93		5				5
	GH95		90				90
	GH97		14				14
	GH99		4				4
	GH113	predicted	1				1
	GH114		1				1
	GH115		21				21
	GH120		3				3
	GH125		11				11
	GH127		41				41
	GH130		31			1	32
	GH141	predicted	29				29
	GH148		14				14
	GH149		1				1
	GH152			3	1	4	8
	GH161		12				12
	GH164	predicted	3				3
	GH165	predicted	3				3
Pectinases	PL1		28			1	29
	PL10		1				1
	PL11		2				2
	PL22		1				1
	PL9		16				16
	CE8	predicted	15	3	1	1	20
	CE12		3				3
	CE19		15				15
	GH28		52		1	3	56
	GH33		25				25
	GH78		69				69
	GH105		62				62
	GH106		22				22
	GH127		41				41
	GH137		1				1
	GH138		4				4
	GH139		5				5
	GH140		12				12
	GH141	predicted	29				29
	GH142		1				1
	GH143		2				2
Lignocellulose binding modules	CBM1	Not Applicable (NA)				1	1
	CBM2	NA	4				4
	CBM4	NA	3				3
	CBM6	NA	26				26
	CBM9	NA	32				32
	CBM13	NA	5	1	3	6	15
	CBM16	NA	1				1
	CBM22	NA	25				25
	CBM30	NA	1				1
	CBM35	NA	5				5
	CBM36	NA	3				3
	CBM39	NA				1	1
	CBM41	NA	4				4
	CBM42	NA	1				1
	CBM46	NA	2				2
	CBM47	NA			1		1
	CBM54	NA	88				88
	CBM57	NA	2			1	3
	CBM61	NA	9				9
	CBM62	NA	10				10
	CBM65	NA	2				2
	CBM67	NA	58				58
	CBM77	NA	2				2
	CBM88	NA	1				1

**Fig. 7:**
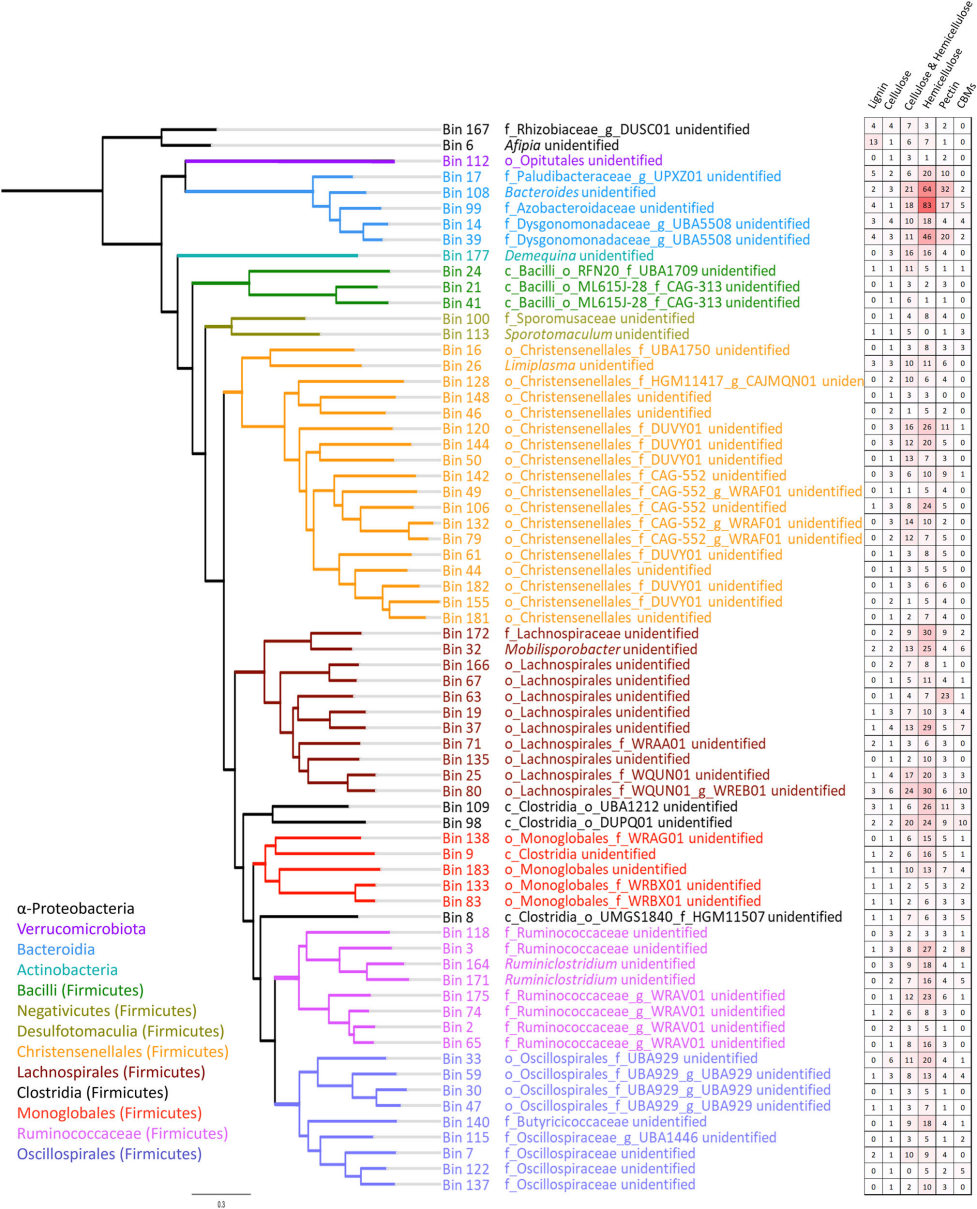
The phylogenetic association of the independent PCWDE providers. The phylogenetic relationship and CAZyme potentials of 68 reassembled metagenome bins with independent cellulase and hemicellulases. Branches and labels with different colors presents for different taxonomic clades. Taxonomic level: c_, class; o_, order; f_, family. The heatmap on the right addresses the number of genes for lignocellulolytic CAZymes, pectinases, and lignocellulose-binding modules in each corresponding bin.

To understand the PCWDE potential of each individual microbe in the microbiome, metagenome bins with independent cellulases and associated lignocellulases were applied to reconstruct the phylogeny of the gut microbiome (Fig. 7). The distribution of ligninases, cellulases, hemicellulases, and pectinases was not limited to a specific group of microbes, though LDMs were mostly found from microbes in the class Clostridia. The second largest phylum providing lignocellulose-degrading enzymes was Bacteroidota. Bins from Actinobacteria, Proteobacteria, and Verrucomicrobiota also contained independent cellulases and hemicellulases.

## Discussion

Previous studies on the CRB microbiome focused on identifying its members, but were unable to characterize the core microbes, divide the enzyme production roles between host and microbes, or identify host structures housing these digestive symbionts and their transmission routes^4,25,26^. Additionally, there were uncertainties about sample size and possible contamination of the isolated microbes^4^, a lack of persuasive implications for the discovery of cellulolytic microbes from CRB^4^, lower accuracy of assigned taxonomies resulting from Illumina short reads compared to 16S full-length PacBio reads^25,26^, and a lack of clear connections and functions between the microbes and CRB^25,26^. This study confirmed high PCWDE activity in the CRB gut, but determined that the beetle is highly dependent on its gut microbes for digestion.

The 16S rRNA gene metabarcoding microbiomes of third instar CRB with different diets and from different locations in Taiwan were compared. Except for an outlier individual, all CRB individuals regardless of diet and location shared the same, top most frequently occurring clades, most of which belonged to class Clostridia, suggesting a mutualistic relationship. Many Clostridia species are known to produce cellulases, including those from the genera identified in this study, such as *Clostridium*, *Acetivibrio*, *Ruminiclostridium*, and *Christensenella*^29–37^. By contrast, the endogenous CRB cellulase did not show any detectable PCWDE activity, despite successful heterologous expression using standard methods, as predicted in the original transcriptome publication due to its low expression levels^25^. The evidence all suggests that CRB uses its gut microbiome as the source of its PCWDEs and has a mutualistic association with a core microbiome comprised primarily of Clostridia, at least in Taiwan. While Clostridia is a cosmopolitan class, such that CRB worldwide probably do live in habitats with some species of Clostridia, the possibility exists that CRB core microbiomes in other parts of the world consist of different microbes, including possibly non-Clostridia. The authors are currently profiling the microbiomes of CRB from other countries to check if their core microbes are the same.

On the other hand, the culturing-dependent microbiomes are significantly different from the culturing-independent metagenomic microbiomes. The incongruencies in microbial composition can also be observed in previous microbiome research^38,39^. The characterization of the hindgut microbial community may be largely affected by isolation methods and DNA extraction methods^38,39^. Since the hindgut of insects is an anaerobic environment, common aerobic culturing methods will not isolate the most abundant strains in the gut, and the cultured strains are likely to be infrequent in the microbiome. Many unculturable strains in turn may be overlooked due to primer affinity. Thus, combining both culturing-dependent and culturing-independent approaches could provide a better and more comprehensive microbiome profile for the target organism.

The correlational network analysis shows that most of the interactions between the core microbes are positive or neutral, indicating the core microbes likely cooperate with each other and establish a symbiotic association with their host together. Thus, CRB forms a symbiotic relationship with a variable microbial community rather than just one, two, or a few specific microbes. This is very different from other digestive symbioses in insects, such as tortoise leaf beetles (Chrysomelidae) that are associated with *Candidatus* Stammera for pectinases^40,41^, desert weevils (Curculionidae) with *Citrobacter* for cellulose degradation and other nutritional related functions^42^, and classic nutritional symbiosis in pea aphids (*Acyrthosphion pisum*) with *Buchnera aphidicola* for essential amino acid provision^43^. CRB’s symbiont community is thus closer in diversity to those of lower termites^44,45^, except lacking their horizontal transfer and maintenance of the symbiotic community.

The prokaryotic metagenome provides a comprehensive profile of PCWDEs, while the host transcriptome has few PCWDE genes of limited types, further suggesting the importance of CRB’s larval microbiome for providing digestive enzymes, especially cellulases. Besides CRB, many other scarab species are known to produce a few PCWDEs, typically GH9 based on their transcriptome or genome^5,6^. However, to date, no confirmation had been made of whether the GH9s from these beetles are active or not. This study found the endogenous GH9 of CRB is inactive against all substrates of GH9, which suggests a similar situation may be found in other, closely related scarabs as well. If that is the case, scarabs generally would need to establish digestive associations with lignocellulose degrading microbes to survive.

The establishment of digestive symbiosis is difficult. The host needs to acquire beneficial microbes and provide a suitable, selective habitat for the desired microbes to reside in. In CRB, a close association between microbes and host tissues is present in the biofilm on the tree-like structures, which can only be observed in the larval hindgut, but not in the adults’; suggesting the larval CRB is more likely to rely on those gut microbes for certain purposes since biofilm formation indicates a stable association of the symbionts and the host^46–49^. Hypothetically, the symbiosis starts when chemical cues from the host trigger chemotaxis in specific groups of bacteria that attach on the gut inner lining. After microbe-microbe competition (selection) for the gut niche, the selected bacteria can avoid triggering the host immunity by adjusting their outer-membrane composition or structure, or evolving certain structures that inhibit host-derived antimicrobial compounds. Once the symbionts colonize the gut, some of them start biofilm formation, providing protection for the symbionts under many stressors, including host antimicrobials^46,49^. The host also regulates the biofilm by an immune response if pathogens are detected, or if mutant symbionts form unstable biofilms^46,47^. Future work will examine the immune pathways and peptides of CRB to determine what environment the biofilms form under.

This study suggests the mother CRB can pass some microbes to her offspring, but few microbes will persist into larvahood. The role of those microbes in CRB is unclear, possibly providing antimicrobials to defend the egg against invasion of entomopathogens^50^. On the contrary, larval CRB harbors diverse and unique microbes that are not detectable in habitat soil or the adult’s digestive tracts. The gut microbes were not detected in the soil, but still could have been present. A possible explanation is that the larvae acquire the microbes horizontally from the habitat, and that the CRB gut selects for the symbiotic strains by providing a more ideal environment for their growth and development, so they gradually dominate in the gut despite being uncommon in the soil. To ensure microbial acquisition from the environment, chemical cues emitted by microbes likely facilitate olfactory communication with insect hosts, thereby maintaining reliable horizontal transmission^51,52^. This phenomenon is evident among immature adults of the Eurasian spruce bark beetles (*Ips typographus*; Curculionidea; Curculionidae; Scolytinae) and several species of dung beetles (Scarabaeoidea). The bark beetle’s association with its symbiotic fungi is facultative and horizontally acquired. It is attracted by the volatiles emitted from the mutualistic fungi to maintain the symbiotic relationship^53–55^. Dung beetles, being the relatives of CRBs in the same superfamily, are known to be attracted to dung volatiles produced during microbial fermentation processes^56,57^. These ecologies may be associated with CRB ecology, as female adult CRBs prefer to oviposit on decaying fibers, which is likely oriented by the olfactory signaling emitted by the environmental microbes, resembling the ecological preferences observed in the aforementioned cases, to ensure the functional benefit of environmental microbial transmission for CRB digestion.

According to the functional profile prediction, the potential PCWDE productions and the accumulative PCWDEs by types among different stages are similar, which suggests that even in different stages with different microbiomes, the PCWDEs produced are the same. The adult CRB digestive tracts have relatively monotonous and less diverse microbiomes compared to the larval ones, which is expected as the adults feed primarily on sap or fruit and are less dependent on PCWDEs for survival. Similar phenomena have been observed in the dung beetle *C. incertus* and the Japanese beetle *P. japonica*^12,13^. These situations prevent vertical transmission, as the larval microbiome is not retained during metamorphosis, which occurs in the larval habitat.

A related hypothesis is that, in different developmental stages, the gut microbiome may serve different functions. In the early instar larva, the microbiome may be critical for defending the gut against pathogenic microbes through competitive exclusion, at least until the larval immune system matures. Afterwards, the core microbes would provide mostly digestive services until pupation. Since the oviposition location is always uncertain and likely contains diverse environmental microbiomes, it would be evolutionarily advantageous for CRBs to pass their core microbes to the next generation to enhance the survivorship, but this does not appear to be the case. Alternatively, having a flexible tolerance for different genera as members of the core microbial community, as observed in this study, would eliminate the need for female provisioning of larval microbes, as any number of microbes could be equally advantageous. This flexibility may help explain how the invasive CRB can be widely distributed worldwide and adapt to local environments, since their capability for selecting beneficial microbes from the habitat as digestive symbionts helps them survive and establish almost anywhere.

**Fig. 8:**
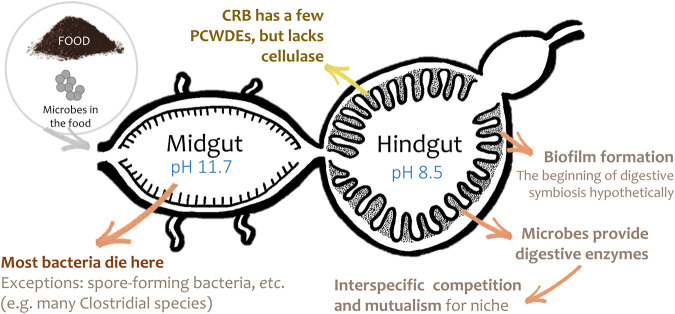
The infographic summary of the digestive symbiosis in CRB. When CRB consumes food, environmental microbes in the food are ingested at the same time. Most bacteria will die in the midgut, an anaerobic and highly alkaline environment at pH 11.7 in CRB and with high immune activity in most insects. Exceptions would be anaerobic bacteria with adaptations for persistence or tolerance, such as the spore-forming Clostridial species, which are cosmopolitan but not relatively abundant in the food. Once these bacteria reach the hindgut, with pH 8.5, some may attach to the inner lining of the gut, multiply greatly, and form a biofilm. This study found multiple taxa common and abundant in the biofilms, known to produce PCWDEs. These conserved core microbes commonly interact with each other mutualistically or neutrally. As CRB produces insufficient PCWDEs to digest their diet, the core microbes provide the necessary enzymes in exchange for a favorable environment.

In summary, the core microbiomes of the larval CRB in this study were mainly comprised of Clostridial species probably acquired and enriched from the environment that assist in the digestive process by producing PCWDEs that the host lacks (Fig. 8). Further studies localizing those microbes in the gut by fluorescence in situ hybridization are necessary to confirm their presence in the hindgut biofilm and better understand this symbiosis. Performing similar work in other Scarabaeidae can provide information on how these insect-microbe interactions evolved.

## Methods

This study was approved to performed at Insect Microbiology Lab at National Taiwan University, Taipei, Taiwan, in accordance to the Environmental Safety and Health Manual of National Taiwan University and Occupational Safety and Health Act.

### Beetle collection, rearing, and dissection

CRBs were collected from several locations in Taiwan (Supplementary Table 3, Supplementary Fig. 3). The beetles for microbiome analyses were dissected within 24 h after collecting them, and the rest of the live beetles were reared in plastic insect boxes with commercial beetle-rearing soil to establish the population in the lab.

For the microbiome profiling regarding different stages of development, CRBs were collected from Pingtung in Taiwan and reared in an incubator at 28 °C to maintain and establish the population in the lab.

For dissection, the beetle samples were chilled at −20 °C individually for 10 min to anesthetize them, then soaked in 75% ethanol in a 50 mL sterile falcon tube and washed by inverting the tube for 2 min to sterilize them. Dissection was conducted in a laminar flow hood with sterile tools.

### Determination of the activity of plant cell wall degrading enzymes by chemical analysis of beetles’ food and feces

To determine the proportion of cellulose, lignin, pectin, and other wood constituents in the starting cocopeat (decayed coconut husks in powder form) that CRB is feeding on and feces collected from wild caught CRB under lab rearing environment, 150 g of cocopeat and 150 g third instar feces, were successively extracted with 75% ethanol, then 100% acetone, then air-dried. These air-dried samples were ground to 40–60 mesh sample meals. These sample meals were further Soxhlet-extracted with toluene/ethanol (2/1, v/v) for one full day, and with 100% ethanol for another full day to remove the extractives. The sample meals were vacuum-dried over P_2_O_5 (s)_ to remove all trapped water within the meals. Lignin content was analyzed by the wet chemistry method-Klason lignin method^58^, combining both the acid-insoluble lignin and acid-soluble lignin as the total lignin content. The extinction coefficient was 110 l g^−1^cm^−1^ at 205 nm^58^. Neutral sugar composition was determined as alditol acetates from the acid-soluble fraction of lignin determination^59^, and quantified by a gas chromatography equipped with a flame ionization detector (GC-FID, Agilent 7890 A). The contents of uronic acid were determined by the carbazole method^60^. Accordingly, 0.5 ml of the acid-soluble fraction from the lignin determination was mixed with 3 ml sulfuric acid reagent (0.025 M sodium tetraborate·10H_2_O in H_2_SO_4_), boiled for 10 min and cooled to room temperature. Then 0.1 ml of carbazole reagent (0.125% carbazole in ethanol) was added to this solution, further boiled for 15 min and cooled to room temperature. Glucuronolactone was used as a standard and the optical density was collected at 530 nm.

To better verify the lignin levels, 0.15 g of cocopeat and third instar feces were sent for nuclear magnetic resonance (NMR) analysis using a Bruker AVANCE III-400MHz Solid-state ^13^C NMR spectrometer at the Instrumentation Center at National Tsing Hua University (Hsinchu, Taiwan), operating at a ^13^C frequency of 100 MHz with standard ramp cross-polarization magic angle spinning (CPMAS) spectroscopy. The powder sample was packed into a 4 mm diameter zirconia rotor and spun at a speed of 10 kHz. A contact time of 3 ms and a pulse delay of 1 s were used for all spectra, and 10,000 scans were accumulated.

### Determination of the activity of plant cell wall degrading enzymes by digestome activity for the substrates

To test for digestive enzymes, including any endogenous or microbial PCWDEs, the midgut and hindgut lumens were rinsed to remove gut contents. The midgut was rinsed with Tris-HCl buffer at pH 10 (approximating the midgut’s alkaline pH), and the hindgut was rinsed with Tris-HCl buffer at pH 8 (approximating the hindgut’s mild alkaline pH). Samples were collected from three third-instar larvae, three pupae, and three adults from lab-reared culture. The pH of the samples was measured using Hydrion® pH strips (Micro Essential Laboratory Inc., USA). The samples were macerated in 100 mM citrate phosphate buffer (pH 5.0) with a dissolved tablet of protease inhibitor cocktail tablet (Roche). The supernatant was collected after centrifugation and applied to plate assays for testing PCWDE activity. Plate assays were performed using carboxymethylcellulose (CMC, Fisher Scientific), xylan from corn core (Tokyo Chemical Industry), xyloglucan from tarmarin seed (Megazyme), glucomannan (Megazyme), and galactomannan (Megazyme) agars at different pHs (0.1% polysaccharide substrate, 0.4% agarose, 50 mM citrate phosphate buffer (CPB) for pH 5–7.0, and Tris-HCl buffer for pH 8–10). Small wells were made using a pipette tip with the end cut off, and 5 µL of enzymes or positive (cellulase from *Aspergillus niger*, Tokyo Chemical Industry) and negative controls (Tris-HCl pH 8.0) were placed in the wells. After the wells dried, the plates were put upside-down into a ziplock plastic bag with a wet paper towel to keep them humid and incubated for 3 h at 40 °C. Subsequently, the plates were stained with 0.1% Congo Red for 60 min and destained with 1 M NaCl_(aq)_ for another 60 min. Finally, the plates were photographed with a light box. If a clear halo appeared around the wells, there was enzyme activity^8,61^.

### In vivo PCWDE expression and test for substrate activity

Shelomi et al. had performed a transcriptome analysis of CRB larvae and found a complete cellulase gene (Accession number: MN047310)^25^. To express this gene as a protein, the total RNA of midgut tissues from the third instar larvae were extracted using TRIzol^Ⓡ^ Reagent (Ambion). RNA was purified first with DNase treatment (TURBO^TM^ DNase, Thermo Fisher Scientific), and then the RNeasy MinElute Cleanup Kit (Qiagen, Hilden, Germany) following the manufacturer’s instructions. Complementary DNA (cDNA) was synthesized using SuperScript III First-Strand Synthesis SuperMix (Thermo Fisher Scientific) following the manufacturer’s instructions. cDNA was amplified for downstream cloning, with specific forward (Orhi_GH9_F: 5′-GCC ACC ATG GAG ATG AAA TAT TTC ATC CAC-3′) and reverse (Orhi_GH9_R: 5′- TTC GGT TTG ACT CTC TAC TTC G-3′) primer-pairs designed to amplify the complete cellulase, with a Kozak sequence included at the 5′ end of the forward primer and no stop codon in the reverse primer.

Each PCR reaction contained 2.5 µL of 10X AccuPrime™ PCR Buffer II (Invitrogen™), 0.2 µL of AccuPrime™ Taq DNA-Polymerase (Invitrogen™), 17.8 µL of nuclease free water (Invitrogen™), 1 µL of forward primer, 1 µL of reverse primer, and 2.5 µL of cDNA. PCR was carried out using the following conditions: denaturation at 95 °C for 2 min; 30 cycles of denaturation at 95 °C for 20 s, annealing at 64 °C for 30 s and extension at 68 °C for 2 min; a final extension at 68 °C for 10 min; and holding at 4 °C.

The resulting amplicons were first checked by gel electrophoresis using 1.5% agarose gel. Once a band in size of approximately 1410 bp was observed, it was cut and the PCR product purified using Zymoclean™ Gel DNA Recovery Kit (ZYMO Research) and cloned into Top10 cells with the pIB/V5-His TOPO^Ⓡ^TA Expression^Ⓡ^ Kit (Invitrogen). Then, colony PCR with the forward primer from the insert and the reverse primer from the vector (OpIE2-R) was done to ensure the target gene was cloned in the right direction, and the plasmids extracted with a GeneJET™ Plasmid Miniprep Kit (Thermo Scientific), sent for Sanger sequencing to check the gene was cloned correctly, and transfected into *Sf9* cells (Invitrogen) using the reagent FuGENE HD (Promega). Cell culture was centrifuged to harvest the enzyme after 72 h incubation at 27 °C, and the resulting supernatant was tested for successful expression via Western Blot with anti-V5-HRP antibody (Invitrogen)^8^. The raw and unprocessed result of this Western Blot was displayed in Supplementary Fig. 4.

Plate assays for enzyme activity were performed on petri dishes of CMC agar (0.1% CMC, 0.4% agarose, solution buffer pH from 5–9), with the detailed recipe for solution buffer shown in Supplementary Table 1. To complement the results of the plate assays, the cellulase was desalted using ChromoTek V5-Trap® Magnetic Agarose (ChromoTek GmbH, Germany), according to the manufacturer’s instructions, and preserved at 4 °C until use. To ensure the successful purification process, Western blot of the enzyme after pull-down assays (desalted process of cellulase) for thin layer chromatography (TLC) assays was performed. The transfectant of CRB’s cellulase with protein expression in the size of approximately 65 kDa suggesting a successful purification process. During the purification processes, no cellulase was detected in the first flow-through during the purification process and the flow-through during the wash step in the purification process, indicating the recovery of the protein was efficiently processed. The raw and unprocessed result of this Western Blot was displayed in Supplementary Fig. 5. Ten microliters of desalted enzyme were combined in microcentrifuge tubes with 1% w/v polysaccharide substrate in ddH_2_O and 0.2 M CPB (pH 5) with a total volume of 20 mL to reach different pH’s as described in Supplementary Table 4. The enzyme-substrate mixtures were incubated overnight at 40 °C, then spotted onto TLC plates (silica gel 60, 20 × 10 cm, Merck) and developed with 9:3:1:4 of ethyl acetate: acetic acid: formic acid: ddH_2_O. Reference standards for the cellulosic substrates and xyloglucan were 2 mg each of glucose, cellobiose, cellotriose, cellotetraose, cellopentaose, cellohexaose, xylose, xylobiose, xylotriose, xylotetraose, xylopentaose, xylohexaose, and D-mannose. For xylan and xyloglucan, reference standards were 2 mg each of xylose, xylobiose, xylotriose xylotetraose, xylopentaose, and xylohexaose. For mannans and galactomannan, the reference standards included glucose standards and 2 mg each of D-mannose, mannobiose, mannotriose, mannotetraose, mannopentaose, mannohexaose and galactose. The detailed recipe is in Supplementary Table 5. Positive controls were cellulase from *Trichoderma reesei* ATCC 26921 (Sigma Aldrich) and cellulase expressed from *Cassida rubiginosa*. The dried plates were sprayed with 0.2% (w/v) orcinol in 9: 1 methanol: sulfuric acid using a CAMAG® Derivatizer (CAMAG Germany), then warmed with a heat gun until spots appeared^61^.

### Determination of the activity of plant cell wall degrading enzymes by isolated microbes of CRB

Microbes isolated from midgut contents and hindgut contents were tested with plate assays for the determination of plant cell wall degradability in vitro. Procedures of microbial isolation are discussed in later paragraphs. Carboxymethylcellulose agar (CMC agar, 1% CMC, 0.2% NaNO_3,_ 0.1% KH_2_PO_4,_ 0.025% MgSO_4_ anhydrous, 0.01% CaCl_2 *_ 2H_2_O, 0.1% Yeast extract & 1.5% Agar at final pH 7.0) and xylan agar (1% xylan, 0.2% NaNO_3,_ 0.1% KH_2_PO_4,_ 0.025% MgSO_4_ anhydrous, 0.01% CaCl_2 *_ 2H_2_O, 0.1% Yeast extract & 1.5% Agar at final pH 7.0) were used for cellulolytic and xylanolytic activity tests with Congo red staining. Three single colonies of the same strain were placed on the agar plate and cultivated for one day on CMC agar or two days on xylan agar at 30 °C. Before staining, the diameter of the single colonies was measured. Subsequently, the plate was put on the shaker and flooded to cover the surface with 0.1% Congo red (in 0.1 M Tris-HCl at final pH 8.0), and shaken for 60 min at 20 rpm. The stain was poured from plates into a liquid waste container and the plates flooded with 1 M NaCl and shaken for 1 h or more. Then, the waste was poured from the plates into the liquid waste container. The plates were photographed with a light box. If a clear halo around the colony appeared, then there was enzyme activity. The diameter of the halo was measured and compared with that of the colony itself.

PT medium (0.5% polygalacturonic acid, 0.1% NaNO_3,_, 0.4% K_2_HPO_4_, 0.02% MgSO4, 0.01% tergitol 7, 1.8% Agar at final pH 7.0) was used for the pectinase activity test. Three single colonies of the same strain were placed on the agar plate and cultivated for two days and afterwards the surface flooded with 1% cetyltrimethyl ammonium bromide (CTAB). A clear halo appeared if the microbe can degrade polygalacturonic acid.

### Scanning electron microscopy for the symbiotic structures

Photos by WHITED CS-3 dissecting microscope had revealed that tree-like structures in the larval hindgut (Fig. 2a, b) are not present in the adult hindgut, which were hypothesized to provide attachment points for symbiotic microbes. Scanning electron microscopy (SEM) was used to observe these structures in more detail. Midgut and hindgut tissue samples from third instar larvae and adults were dissected and rinsed with 0.1 M phosphate buffered saline (PBS, pH7.4). The hindgut tissues were then fixed in 2.5% glutaraldehyde for two days at 4 °C and postfixed in 1% osmium tetroxide solution, then washed three times for 10 min with 0.1 M PBS. After being dehydrated in a graded ethanol series (35%, 35%, 50%, 60%, 70%, 85%, 90%, 95%,100%, 100%, 100%) samples were critical point dried using Leica EM CPD300 Critical Point Dryer, and gold coated in SPI Sputter Coater. Finally, samples were analyzed and photographed at the Joint Center for Instruments and Researches, College of Bioresources and Agriculture, National Taiwan University, using the Jeol JSM-6510LV scanning electron microscope^15,17^.

### DNA purification for profiling the microbiome of larvae around Taiwan

In total, 40 third instar larvae were dissected for 16 S rRNA gene metabarcoding microbiome analysis around Taiwan (Supplementary Table 3, Supplementary Fig. 3).

The hindgut tissues and hindgut contents were isolated and subjected to DNA extraction and purification. The DNA from the hindgut tissues was extracted using EasyPure Genomic DNA Reagent (Bioman), and the DNA from the hindgut contents was extracted using the Presto^TM^ Soil DNA Extraction Kit (Geneaid), according to the manufacturer’s instructions. The extracted DNA was prepurified using the OneStep PCR Inhibitor Removal Kit (ZYMO Research), then purified with the DNeasy® PowerClean® Pro Cleanup Kit (QIAGEN).

### Microbe isolation and identification

In total, 44 third instar larvae and 7 s instar larvae were dissected for microbe isolation of culturing dependent microbiome around Taiwan (Tables S3, S6).

Microbes were isolated from midgut contents and hindgut contents by streak plate method on nutrient agar (NA; HIMEDIA) for bacteria and potato dextrose agar (PDA; HIMEDIA) supplemented with 100 ppm of chloramphenicol for fungi. After three rounds of sub-culturing, single colonies were selected for cell lysis by boiling or DNA extraction using EasyPure Genomic DNA Reagent (Bioman), and used for 16 S *rRNA* (27 F: 5′-AGA GTT TGA TCM TGG CTC AG-3′; 338 F: 5′-ACT CCT ACG GGA GGC AGC AG-3′; 1492 R: 5′-CGG TTA CCT TGT TAC GAC TT-3′), *rpoB*^62^ (*rpoB*1206: 5′- ATC GAA ACG CCT GAA GGT CCA AAC AT-3′; *rpoB*3202: 5′-ACA CCC TTG TTA CCG TGA CGA CC-3′) to amplify barcoding genes for bacteria, and/or ITS rDNA (ITS1F: 5′-CTT GGT CAT TTA GAG GAA GTA A-3′;ITS4R: 5′-TCC TCC GCT TAT TGA TAT GC-3′) for fungi.

Each PCR reaction was contained 12.5 µL of 2X Taq PCR Mix-RED (Bioman), 10.5 µL of nuclease free water (Bioman), 0.5 µL of forward primer, 0.5 µL of reverse primer and 1 µL of lysed DNA. The PCR reaction for 16 S *rRNA, rpoB*, and ITS rDNA were run with the following cycling programs: for the 16 S rRNA and ITS rDNA, denaturation was started at 94 °C for 3 min, 35 cycles of denaturation at 94 °C for 45 s, annealing at 55 °C for 60 s, and extension at 72 °C for 90 s, a final extension at 72 °C for 5 min, and held at 4 °C. For the *rpoB*, denaturation was started at 95 °C for 3 min, 35 cycles of denaturation at 95 °C for 20 s, annealing at 55 °C for 30 s and extension at 72 °C for 90 s, a final extension at 72 °C for 5 min and held at 4 °C. The annealing temperature varied and needed to be adjusted slightly for different samples.

Gel electrophoresis was conducted to check if the target gene was amplified. If a band in size of approximately 1.5 Kb (16 S *rRNA* and *rpoB*) or 550 bp (ITS region) formed, the corresponding PCR product was purified using an EasyPure PCR/Gel Extraction Kit (Bioman). The purified PCR products were sent to the DNA Sequencing Core of Center for Biotechnology at National Taiwan University for sequencing.

The resulting sequences were firstly de-novo assembled from forward and reverse sequences and the ambiguous ends removed with Geneious Prime, aligned with MUSCLE^63^, and then compared to known microbial sequences on the NCBI database (https://www.ncbi.nlm.nih.gov/) with BLAST.

### Experimental setup and DNA purification for microbiome analysis across different developmental stages

In total, three female adults, 18 eggs, nine 1st instar larvae, nine 2nd instar larvae, and nine 3rd instar larvae from Wandan (WDc) were applied for profiling the microbiome in different stages of development. To see whether the core microbiome of the third instar larvae would be passed to adulthood and transmitted to the offspring, microbiome profiling was done for different developmental stages. The experimental setup is shown in Supplementary Fig. 6.

Female adults with fertile eggs were used for the experiment. For the adult, the midgut and hindgut were used for microbiome analysis. To distinguish between possible routes for vertical transmission of symbionts either on the surface of the egg shell or inside the egg (embryotic transmission), or acquisition from the beetle soil, eggs from the same mother were first divided into two groups, one treated with ddH_2_O as control group, while another was surface sterilized with 1% bleach for 1 min, then rinsed with ddH_2_O for 1 min three times. The whole eggs were smashed to study the microbiome. For the soil, 25 mg of soil were used. For the first instar larvae, since the size of the hindgut was small, the entire intestinal tract was used for microbiome analysis; while for the second and third instar larvae, only the hindgut was used. Three individuals were used for each group or instar of larvae, with three biological replicates. DNA extraction of insect tissues and soil samples were performed using DNeasy Blood & Tissue Kit (QIAGEN).

### Microbiome analysis and functional profile prediction of larvae around Taiwan

The purified DNA samples of the hindgut contents were sent to BioTools Co., Ltd. for 16 S ribosomal DNA sequencing using PacBio Sequel Ile for Q30 HiFi reads. Following sequencing, QIIME 2 (v2022.8) was used for quality filtering, dada2 denoising^64^, and microbiome analysis of the resulting amplicon sequence variants (ASV), including taxon identification and correlating microbial diversity with location and diet^65^. An NCBI RefSeq classifier for prokaryotic 16 S *rRNA* was generated using plugin *RESCRIPt*^66–68^ in python.

Core microbial communities (CCs) were defined as taxonomic clades that appear in at least 90% of all individuals^69^. To determine whether different diets and collecting sites would affect the relative abundance of CCs, Kruskal–Wallis test and the pairwise Wilcox test were conducted with the package *dplyr*^70^ in R. Beta diversity was compared between samples from different locations and diets using non-dimensional scaling (NMDS) by Bray–Curtis distance using the packages *vegan*^71^ and *pairwiseAdonis*^72^ in R with both group and pairwise comparisons (PERMANOVA) and plotting using the *ggplot2* package in R^73^.

To identify the possible association between the core microbes and the transient microbes (defined as microbes found in more than 50% but less than 90% of all sampled individuals), the co-occurrence network of filtered ASVs was calculated using Spearman rank correlation coefficient with the packages *corrplot*^74^, *rstatix*^75^, and visualized with the package *igraph*^76^ in R. Only the correlation coefficient indices (*R* values) with significant *p*-values (*p* < 0.05), which indicates strong correlation) were used for the construction of the network co-occurrence.

### Microbiome analysis and functional profile prediction of microbial dynamics across different developmental stages

The purified DNA samples of the hindgut content were sent to BioTools Co., Ltd. for 16 S ribosomal DNA sequencing using PacBio Sequel Ile for Q30 HiFi reads. The subsequent microbiome, diversity, and taxonomic analyses were performed as aforementioned. To predict possible metabolic functions of the microbiome, especially PCWDE production, Phylogenetic Investigation of Communities by Reconstruction of Unobserved States version 2 (PICRUSt2) was used to perform the functional diversity analysis for each individual sample^77^. Tables containing the predicted gene family-counts based on the Enzyme Commission (EC) were generated for downstream analysis^78^. The functions not related to PCWDE production were removed, and only enzymes belonging to the GH, CE, and PL families were preserved. The relative abundance of total potential PCWDE production by types of enzymes were tested for differential expression by Kruskal–Wallis and pairwise Wilcox tests using the package *dplyr*^70^ in R. Beta diversity based on the PICRUSt2 EC result was compared between samples from different stages of development using NMDS by Bray–Curtis distance with the packages *vegan*^71^ and *pairwiseAdonis*^72^ in R with both group and pairwise comparisons (PERMANOVA) and plotting using the *ggplot2* package in R^73^.

### Whole genome shotgun sequencing, metagenome assembly and annotation, and reanalysis of transcriptome

Since the assigned taxonomies from NCBI RefSeq could not always confirm the identities of many microbial ASVs among the CCs, the functional contribution from the CCs to the host derived from the 16S rRNA gene metabarcoding data contained uncertainties. To better characterize the functional entities of unidentified microbial ASVs in the CCs, especially when many of them are likely to be undescribed species and most of them are unculturable, whole genome shotgun sequencing for prokaryotes was needed. One DNA sample of third instar larval hindgut content purified by using a QIAamp® DNA Micro Kit was sent for Illumina meta-whole genome shotgun sequencing conducted by the DNA Sequencing Core of the Center for Biotechnology, National Taiwan University, on an Illumina NovaSeq 6000 platform.

The resulting sequencing depth was greater than 50 Gb. Raw reads were analyzed following MetaWRAP pipelines described in 2018^79^. Taxonomic identities were assigned to the reassembled bins using GTDB-Tk^80^. The annotations and prediction of different types of lignocellulases and pectinases for each bin were searched for carbohydrate-activated enzymes (CAZymes) modules and assigned by HMMER (*E* < 10^−15^, sequence coverage >0.35) and DIAMOND (*E* < 10^−102^) using the dbCAN web server (https://bcb.unl.edu/dbCAN2/)^81^. The bins with independent cellulase and hemicellulase potential were first aligned by MAFFT^82^ in Geneious v.2023.1.1 and inferred to a maximum likelihood phylogenetic tree constructed by IQTREE web server version 1.6.12 (http://iqtree.cibiv.univie.ac.at/)^83^ with 1000 bootstrap replicates, with the LG + I + G4 substitution model chosen according to Bayesian information criteria identified as the best-fit model by ModelFinder^84^.

To compare these results with the annotations and prediction of different types of lignocellulases and pectinases from the host CRB, previous transcriptome data (the NCBI Short Reads Archive, Accession Numbers: SRR9208133–40) from ref. ^25^ was reanalyzed using the dbCAN web server as well, following the aforementioned procedures. The predicted cellulases from the transcriptome were further checked with their translation to protein sequence using Expasy web server (https://web.expasy.org/translate/) and re-blasted on NCBI protein BLAST (https://blast.ncbi.nlm.nih.gov/Blast.cgi?PAGE=Proteins) to ensure the predicted enzymes are cellulase candidates.

### Reporting summary

Further information on research design is available in the Nature Research Reporting Summary linked to this article.

### Supplementary information

Supplementary Information

Reporting Summary

## Data Availability

The datasets generated and/or analyzed during the current study are available under the BioProject PRJNA997560 in the NCBI GenBank, [https://www.ncbi.nlm.nih.gov/bioproject/PRJNA997560]. All relevant data are available from the authors.
