# Author Correction: Coconut rhinoceros beetle digestive symbiosis with potential plant cell wall degrading microbes

**DOI:** 10.1038/s41522-024-00519-3

**Published:** 2024-05-23

**Authors:** Chiao-Jung Han, Chih-Hsin Cheng, Ting-Feng Yeh, Yannick Pauchet, Matan Shelomi

**Affiliations:** 1 Department of Entomology, National Taiwan University, Taipei, Taiwan; 2 School of Forestry & Resource Conservation, National Taiwan University, Taipei, Taiwan; 3 Department of Insect Symbiosis, Max Planck Institute for Chemical Ecology, Jena, Germany

**Keywords:** Symbiosis, Microbial ecology

Correction to: *npj Biofilms and Microbiomes* 10.1038/s41522-024-00505-9, published online 30 March 2024

In this article, the grant numbers 111L7822, 112L7814, and 113L7801 relating to the ‘Taiwan Ministry of Education’ were omitted. Additionally, a few supplementary table numbers were mislabeled. The original article has been corrected.

