# Supplementary material for: Coconut rhinoceros beetle digestive symbiosis with potential plant cell wall degrading microbes
Source: NPJ Biofilms Microbiomes. 2024 Mar 30;10:34. doi: 10.1038/s41522-024-00505-9 (PMC10981690; doi:10.1038/s41522-024-00505-9)
Supplement: Supplementary file 1 — Supplementary Information [file 41522_2024_505_MOESM1_ESM.pdf]

# Supplementary Information

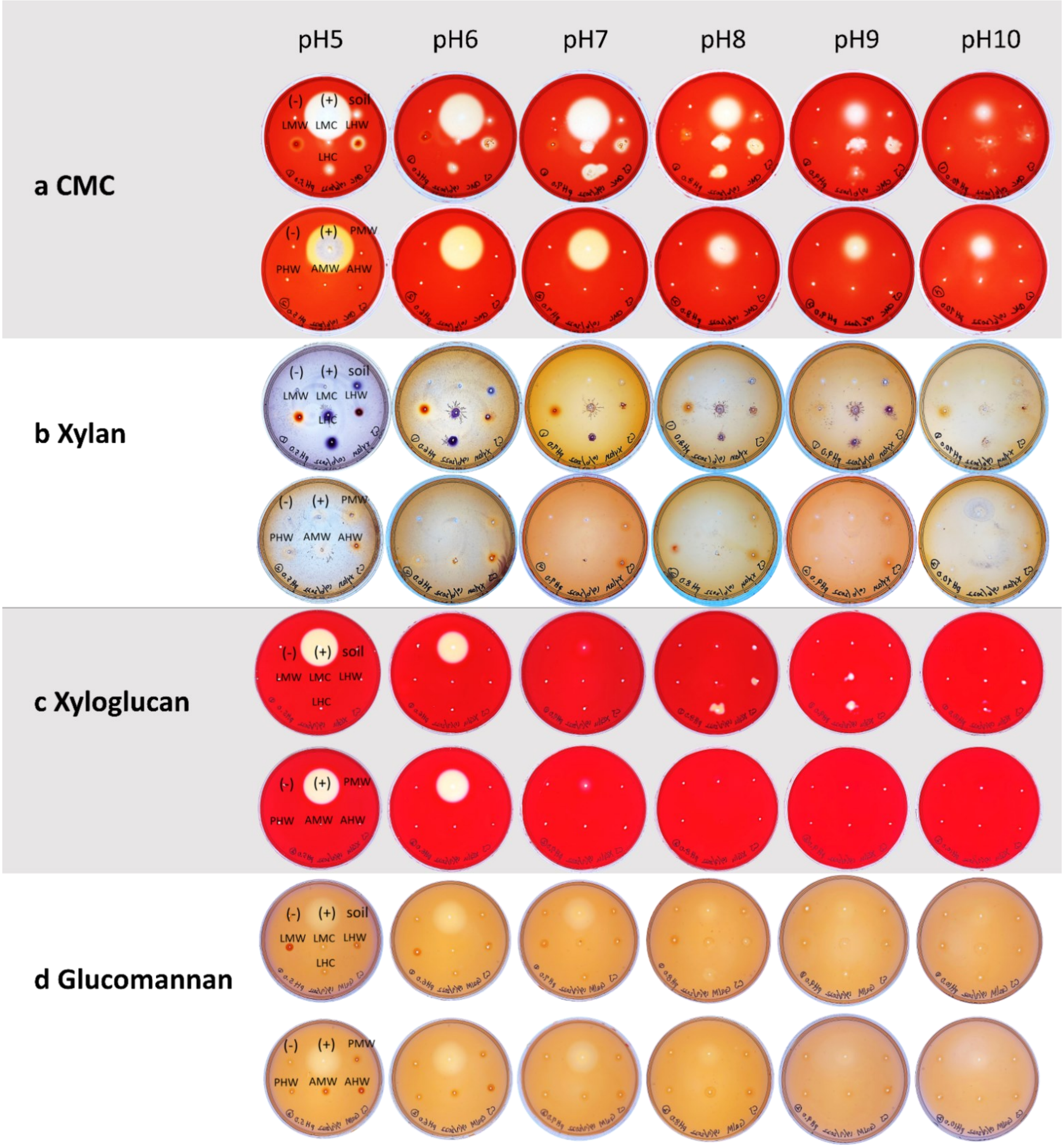


Supplementary Figure 1. Plate assays for testing the digestome activity of four substrates

If a clear halo surrounding the colony is formed, then digestive activity existed against that substrate. (-): buffer as negative control; (+): cellulase from *Aspergillus niger* as positive control; soil: the feeding substrate for the beetles; LMW: larval midgut wall; LMC: larval midgut contents; LHW: larval hindgut wall; LHC: larval hindgut contents; PMW: pupal midgut wall; PHW: pupal hindgut wall; AMW: adult midgut wall; AHW: adult hindgut wall.

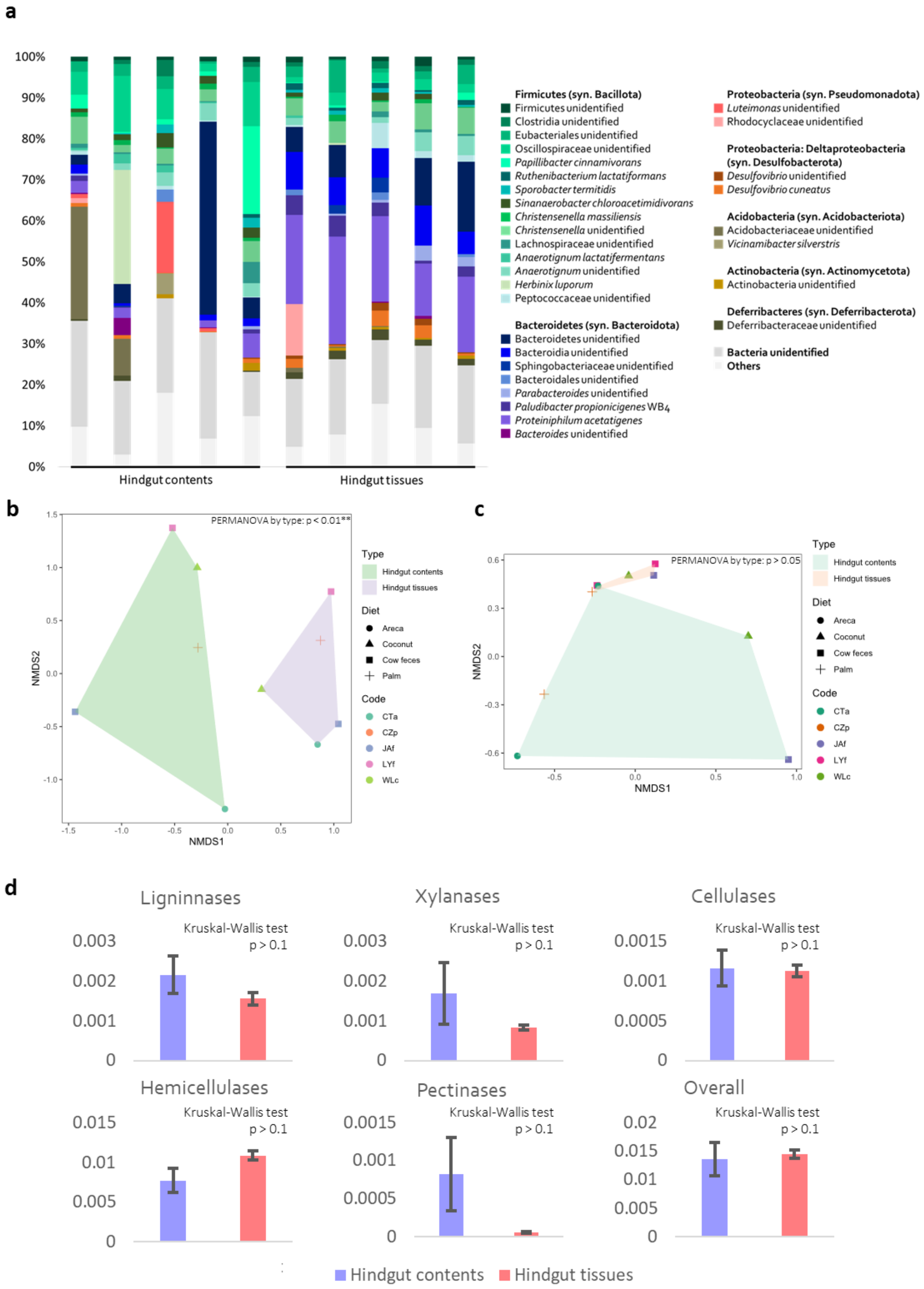


**Supplementary Figure 2. The comparison of the microbiomes and functional profiles between 3rd instar larval hindgut contents and tissues.**

(a) The relative abundance by taxonomic composition among hindgut contents and hindgut tissues from the late larval microbiomes. The details of samples used and the number of PacBio reads were

displayed in Supplementary Table 2. (b) Non-metric multidimensional scaling (NMDS) plot of microbial structures between hindgut contents and tissues by Bray-Curtis distance (Stress = 0.065). A test of significance was conducted using PERMANOVA. Significant differences were found between hindgut contents and tissues ($p < 0.01$). (c) The NMDS plot of the predicted functional profile of the microbiome by Bray-Curtis distance (stress = 0.031). A test of significance was conducted using PERMANOVA. No significant differences were found between hindgut contents and tissues ($p > 0.05$). (d) The relative abundance of the five plant cell wall-degrading enzyme groups predicted according to the PICRUSt2 functional profile analysis. A test of significance was conducted using the Kruskal-Wallis test and pairwise Wilcox test by stadium. The Kruskal-Wallis test and Wilcox test of every group showed no significant differences between hindgut contents and tissues ($p > 0.1$). The error bar represents for the standard error (SE).

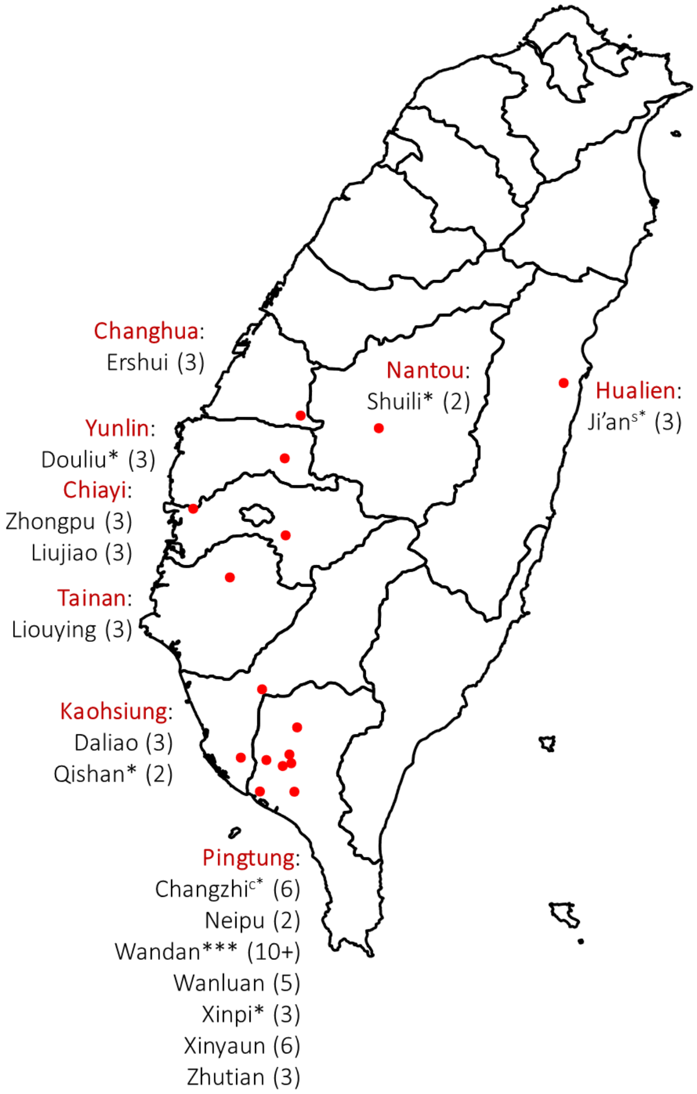


Supplementary Figure 3. CRB sample collection sites around Taiwan

An asterisk in the top right of the code name indicates that these samples were only used for the culturing microbiome, double asterisks indicate that these samples were only used for the metagenomic microbiome, code names without asterisks are samples used for both metagenomic and culturing microbiome analyses, code names with triple asterisks indicates that these samples were reared in laboratory for the metagenomic microbiome of different developmental stages. For the Changzhi samples, only those from coconut were labeled with an asterisk. Similarly, for the Ji’an samples, only those from soil diets were labeled with an asterisk.

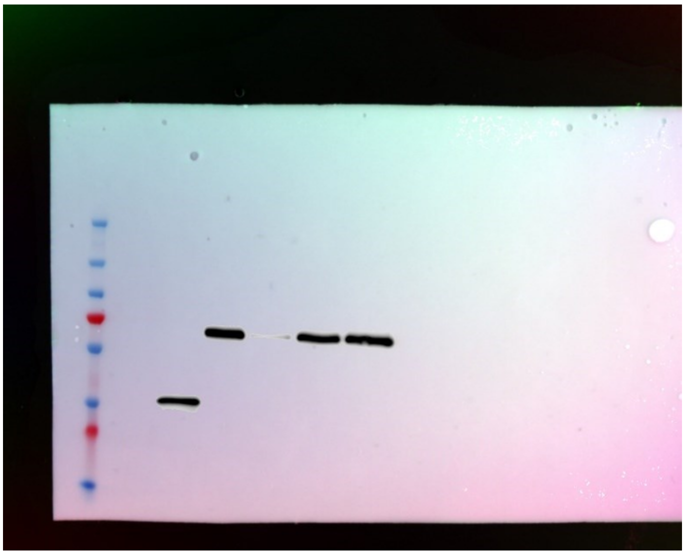

Supplementary Figure 4. The raw and unprocessed Western Blot of the cellulase from CRB expressed by *Sf9* cells as depicted in Figure 2 in the main article.

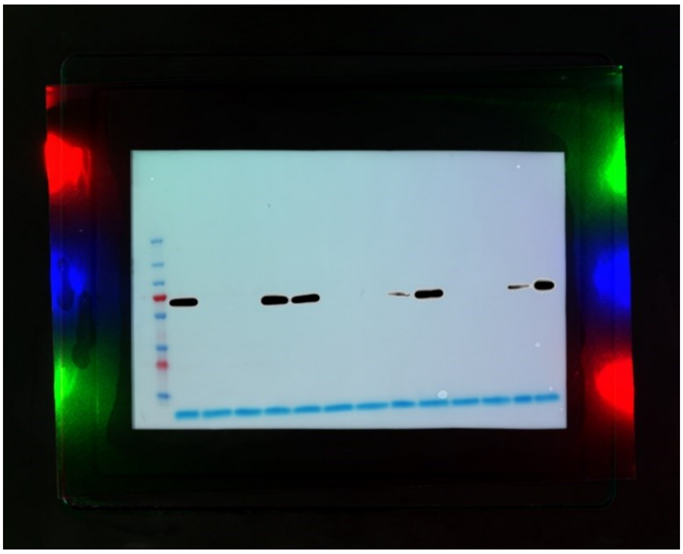

Supplementary Figure 5. The raw and unprocessed Western Blot of the enzyme after pull-down assays for thin layer chromatography (TLC) assays as depicted in Figure 3 in the main article.

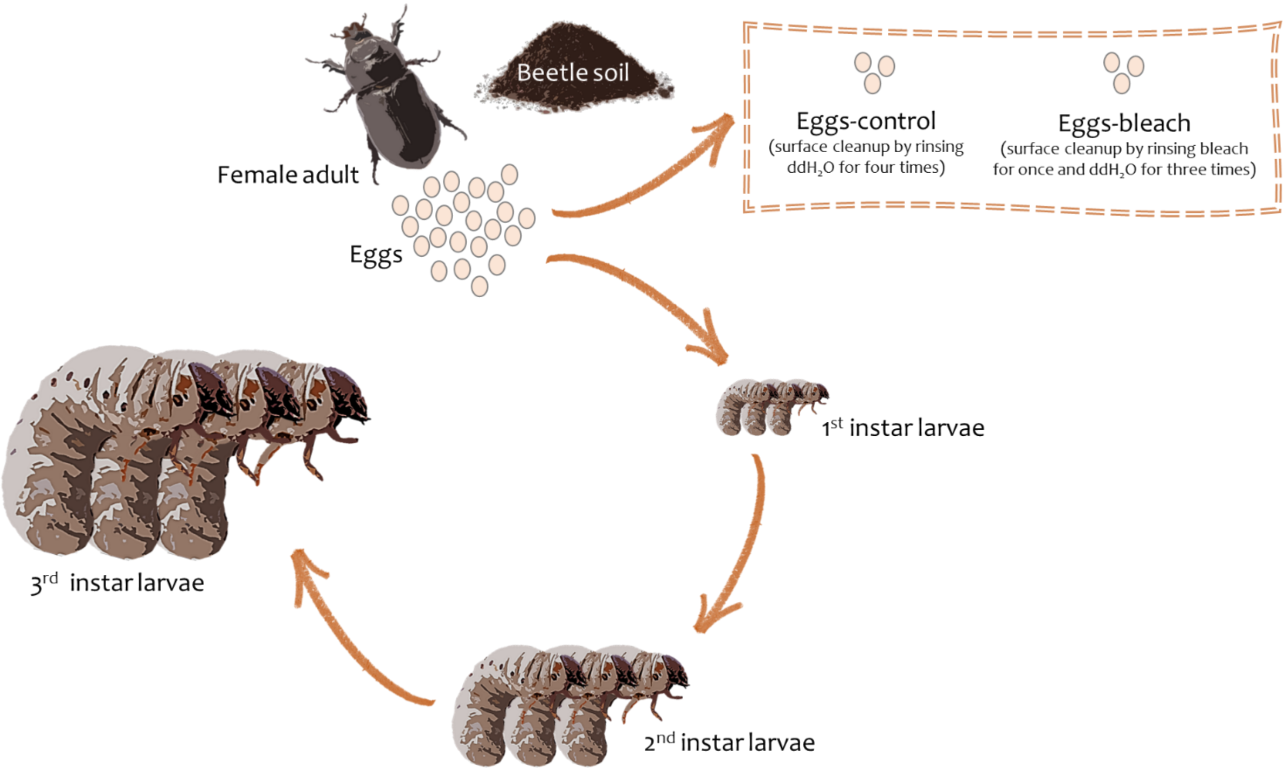


**Supplementary Figure 6. The experimental setup of how we sampled CRB in different developmental stages.**

Female adults with fertile eggs were used for the experiment. For the adult, the midgut and hindgut were used for the microbiome analysis. To distinguish the possible route for vertical transmission of symbionts either on the surface of the egg shell or inside the egg (embryotic transmission), eggs from the same mother were first divided into two groups, one treated with $ddH_2O$ as control group, while another was treated by 1% bleach for one minute, after rinsing using $ddH_2O$ for one minute three times. The whole eggs were smashed to study the microbiome. For the first instar larvae, since the size of the hindgut was small, the whole intestinal tract was used for microbiome analysis; while for the second and third instar larvae, only the hindgut was used. Three individuals were used for each group or instar of larvae, with three biological replicates.

Supplementary Table 1. The PCWDE activity assays by isolated microbes

| Isolates | Species | CMC | Xylanase | Pectinase |
|---|---|---|---|---|
| CTa1L2H2 | *Leminorella grimontii* | - | + | - |
| CTa1L2M1 | *Paraburkholderia caballeronis* | - | + | - |
| CZp1L1H1 | *Klebsiella aerogenes* | +/- | + | - |
| CZp1L2M1 | *Bacillus* sp. | + | + | - |
| CZp1L3M1 | *Bacillus* sp. | + | - | - |
| DLs1L1M1 | *Bacillus* sp. | + | +/- | - |
| LJf1L1M1 | *Bacillus* sp.<br>*Geobacillus stearothermophilus* | + | - | - |
| LJf1L1M2 | *Acinetobacter baumannii* | + | + | - |
| LJf1L3M1 | *Leminorella grimontii* | + | - | - |
| SLa1LM4 | *Staphylococcus aureus* | - | + | - |
| SLa1LH2 | *Bacillus* sp. | + | + | - |
| SLa1LH5 | *Thauera* sp. | + | - | - |
| WLc1L3H1 | *Bacillus* sp. | + | - | - |
| XPs1L3M2 | *Serratia marcescens* | + | - | - |
| XYa1L3H2 | *Edaphobacter* sp. | - | + | - |
| XYa1L5M2 | *Priestia megaterium*<br>*Bacillus* sp.<br>*Priestia aryabhattai* | - | + | - |
| ZPa1L3H1 | *Bacillus* sp. | + | + | - |
| ESs1L3H1 | *Bacillus* sp. | + | - | - |
| ESs1L1H1 | *Bacillus* sp. | + | - | - |
| ESs1L3H3 | *Lysinibacillus macrolides*<br>*Lysinibacillus xylanilyticus*<br>*Bacillus* sp.<br>*Lysinibacillus pakistanensis* | +/- | - | - |
| LYf1L3H1P | *Candida saraburiensis* | + | - | + |
| JAf1L3H1 | *Bacillus* sp. | + | - | - |
| JAf1L2H3 | *Serratia marcescens* | - | - | + |
| JAf1L2H1 | *Bacillus* sp. | + | - | - |
| JAf1L3M1P2 | *Pascua guehoae* | + | + | + |
| DOs1L1H1P | *Candida xylanilytica* | - | + | - |

| | | | | |
|---|---|---|---|---|
| DOs1L1M1 | *Paracoccus* sp. | - | + | - |
| DOs1L1M3 | *Bacillus humi* | - | - | + |
| JAs1L1H2 | *Bacillus* sp. | + | - | - |
| JAs1L1H1P | *Trichosporon siamense* | - | + | - |
| JAs1L2M2 | *Lysinibacillus* sp. | + | - | - |
| JAs1L2M2P | *Trichosporon siamense* | - | + | + |
| JAs1L2M3P | *Candida tropicalis* | - | + | - |
| JAs1L2H1A | *Candida saraburiensis* | + | - | - |

Supplementary Table 2. Details of samples and the number of reads for 16S rRNA gene metabarcoding sequencing.

| Sample group | Code | Stage | Tissue types / treatment | Number of original reads | | | Number of filtered reads | | |
|---|---|---|---|---|---|---|---|---|---|
| | | | | replicate 1 | replicate 2 | replicate 3 | replicate 1 | replicate 2 | replicate 3 |
| Microbial profiling around Taiwan | ESs | 3rd instar larvae | Hindgut contents | 14724 | 14453 | 14704 | 1946 | 1940 | 1706 |
| | ZPa | | | 13329 | 14995 | 16697 | 2953 | 3166 | 4162 |
| | LJf | | | 13562 | 13220 | 14675 | 2681 | 2357 | 2728 |
| | LYf | | | 15061 | 14439 | 14406 | 2671 | 3606 | 4028 |
| | DLs | | | 12593 | 12935 | 14350 | 2969 | 4002 | 3110 |
| | NPc | | | 15634 | 14815 | - | 3515 | 3788 | - |
| | WLa | | | 14254 | 15425 | - | 3184 | 3681 | - |
| | WLc | | | 13399 | 14038 | 15732 | 1789 | 2195 | 2380 |
| | XYa | | | 13361 | 13225 | 15179 | 3697 | 4095 | 4393 |
| | XYc | | | 14508 | 14828 | 13003 | 3781 | 2081 | 1716 |
| | CTa | | | 13271 | 14857 | 14016 | 4295 | 5832 | 4430 |
| | CZp | | | 14114 | 12831 | 13921 | 5449 | 4123 | 1684 |
| | CZc | | | 17458 | 18284 | 18210 | 2624 | 3527 | 3071 |
| | JAf | | | 17614 | 18022 | 16892 | 3859 | 4657 | 3778 |
| Microbiome comparison between hindgut contents and tissues | LYf | 3rd instar larvae | Hindgut contents | 14439 | - | - | 3089 | - | - |
| | WLc | | | 15732 | - | - | 2277 | - | - |
| | CTa | | | 13271 | - | - | 3744 | - | - |
| | CZp | | | 13921 | - | - | 1765 | - | - |
| | JAf | | | 16892 | - | - | 3547 | - | - |
| | LYf | | Hindgut tissues | 10655 | - | - | 2159 | - | - |
| | WLc | | | 19738 | - | - | 3856 | - | - |
| | CTa | | | 10920 | - | - | 2642 | - | - |
| | CZp | | | 10074 | - | - | 2706 | - | - |
| | JAf | | | 12390 | - | - | 2532 | - | - |
| Stages of development | WDc | Female adults | Midgut tissues | 35014 | 24597 | 18776 | 24628 | 9888 | 10947 |
| | | | Hindgut tissues | 43773 | 24888 | 18422 | 13666 | 10254 | 6074 |
| | | eggs | Bleach treated | 28338 | 20487 | 17790 | 20506 | 12755 | 2534 |
| | | | $ddH_2O$ treated | 25018 | 25748 | 18681 | 14406 | 7415 | 3836 |
| | | 1st instar larvae | Whole gut tissues | 19591 | 20698 | 17383 | 4268 | 1989 | 2164 |
| | | 2nd instar larvae | Hindgut tissues | 25936 | 38736 | 22141 | 9876 | 20605 | 11514 |
| | | 3rd instar larvae | | 20347 | 29297 | 22716 | 5478 | 13793 | 12652 |
| | soil | soil | Environment control | 14025 | - | - | 847 | - | - |

Abbreviations of code number as defined in the Supplementary Table 3.

**Supplementary Table 3. CRB sample collection details for downstream experiments.**

| Administrative divisions | Locations | Habitat (host plant) | Stadium | Number of individuals | Code |
|---|---|---|---|---|---|
| **Nantou** | Shuili (SL) | *Areca catechu* (betel palm) | $2^{nd}$ instar | 2 (pooled) | SLa* |
| **Changhua** | Ershui (ES) | *Areca catechu* | $3^{rd}$ instar | 3 | ESs |
| **Yunlin** | Douliu (DO) | Beetle soil (lab reared) | $3^{rd}$ instar | 3 | DOs* |
| **Chiayi** | Zhongpu (ZP) | *Areca catechu* | $3^{rd}$ instar | 3 | ZPa |
| **Chiayi** | Liujiao (LJ) | Cow feces | $3^{rd}$ instar | 3 | LJf |
| **Tainan** | Liuying (LY) | Soil + cow feces | $3^{rd}$ instar | 3 | LYf |
| **Kaohsiung** | Daliao (DL) | *Cocos nucifera* (coconut) | $3^{rd}$ instar | 3 | DLs |
| **Kaohsiung** | Qishan (QS) | *Cocos nucifera* | $2^{nd}$ instar | 2 | QSc* |
| **Pingtung** | Neipu (NP) | *Cocos nucifera* | $3^{rd}$ instar | 3 | NPc |
| **Pingtung** | Wandan (WD) | *Cocos nucifera* | Adults, eggs, and larvae | 48 | WDc*** |
| **Pingtung** | Wanluan (WL) | *Areca catechu* | $3^{rd}$ instar | 2 | WLa |
| **Pingtung** | Wanluan (WL) | *Cocos nucifera* | $3^{rd}$ instar | 3 | WLc |
| **Pingtung** | Xinyuan (XY) | *Areca catechu* | $3^{rd}$ instar | 3 | XYa |
| **Pingtung** | Xinyuan (XY) | *Cocos nucifera* | $3^{rd}$ instar | 3 | XYc |
| **Pingtung** | Zhutian (CT) | *Areca catechu* | $3^{rd}$ instar | 3 | CTa |
| **Pingtung** | Changzhi (CZ) | *Phoenix loureiroi* (palm) | $3^{rd}$ instar | 3 | CZp |
| **Pingtung** | Changzhi (CZ) | *Cocos nucifera* | $3^{rd}$ instar | 3 | CZc** |
| **Pingtung** | Xinpi (XP) | Straw + beetle soil | $2^{nd}$ instar | 3 | XPs* |
| **Hualien** | Ji'an (JA) | Beetle soil (lab reared) | $3^{rd}$ instar | 3 | JAs* |
| **Hualien** | Ji'an (JA) | Soil + cow feces | $3^{rd}$ instar | 3 | JAf |

An asterisk in the top right of the code name indicates that these samples were only used for the culturing microbiome, double asterisks indicates that these samples were only used for the 16S rRNA metabarcoding microbiome, code names without asterisks are samples used for both 16S rRNA metabarcoding and culturing microbiome analyses, code names with triple asterisks indicates that these samples were reared in lab for the 16S rRNA metabarcoding microbiome of different developmental stages.

Supplementary Table 4. Recipe of buffer used in plate assays for substrate activities

| pH | 0.1M Citric acid | 0.2M $Na_2HPO_4$ | 0.1M Tris-HCl |
|---|---|---|---|
| 5 | 4.85 mL | 5.15 mL | 0 |
| 6 | 3.685 mL | 6.315 mL | 0 |
| 7 | 1.765 mL | 8.235 mL | 0 |
| 8 | 0 | 0 | 10 mL (pH 8) |
| 9 | 0 | 0 | 10 mL (pH 9) |
| 10 | 0 | 0 | 10 mL (pH 10) |

Supplementary Table 5. The recipe for thin layer chromatography assays

| Plate 1: using Glu1-6, Xyl1-6 and Man1 as standards | | | Enzyme | substrate | CPB pH5.0 | Merck water | Total volume |
|---|---|---|---|---|---|---|---|
| A | 1 | Carboxymethyl cellulose 4M (CMC4M) | 14 | 4 | 2 | 0 | 20 |
| | 2 | Regenerated amorphous cellulose (RAC) | 14 | 4 | 2 | 0 | 20 |
| | 3 | Cellohexaose (Glu6, 2.5 µg/µL) | 14 | 4 | 2 | 0 | 20 |
| | 4 | Cellopentaose (Glu5, 10 µg/µL) | 14 | 1 | 2 | 3 | 20 |
| | 5 | Cellotatraose (Glu4, 10 µg/µL) | 14 | 1 | 2 | 3 | 20 |
| | 6 | Cellotriose (Glu3, 10 µg/µL) | 14 | 1 | 2 | 3 | 20 |
| | 7 | Cellobiose (Glu2, 10 µg/µL) | 14 | 1 | 2 | 3 | 20 |
| | 8 | Xyloglucan (XGlu) | 14 | 4 | 2 | 0 | 20 |
| B | 1 | Glucomannan (GluM) | 14 | 4 | 2 | 0 | 20 |
| | 2 | Enzyme control | 14 | 0 | 2 | 4 | 20 |
| Plate 2: using Xyl1-6 and Man1-6, and Gal1 as standards | | | Enzyme | substrate | CPB pH5.0 | Merck water | Total volume |
| C | 1 | Xylan beechwood (Xbee) | 14 | 4 | 2 | 0 | 20 |
| | 2 | Xylose hexamer (Xyl6) | 14 | 1 | 2 | 3 | 20 |
| | 3 | Xylose pentamer (Xyl5) | 14 | 1 | 2 | 3 | 20 |
| | 4 | Xylose tetramer (Xyl4) | 14 | 1 | 2 | 3 | 20 |
| | 5 | Xylose trimer (Xyl3) | 14 | 1 | 2 | 3 | 20 |
| | 6 | Xylose dimer (Xyl2) | 14 | 1 | 2 | 3 | 20 |
| | 7 | Galactomannan (GalM) | 14 | 4 | 2 | 0 | 20 |
| | 8 | Mannose hexamer (Man6) | 14 | 1 | 2 | 3 | 20 |
| D | 1 | Mannose pentamer (Man5) | 14 | 1 | 2 | 3 | 20 |
| | 2 | Mannose tetramer (Man4) | 14 | 1 | 2 | 3 | 20 |
| | 3 | Mannose trimer (Man3) | 14 | 1 | 2 | 3 | 20 |
| | 4 | Mannose dimer (Man2) | 14 | 1 | 2 | 3 | 20 |
| | 5 | Enzyme control | 14 | 0 | 2 | 4 | 20 |

For negative controls (plates 3 and 4), the above plates were made using water instead of substrate.

Supplementary Table 6. Identified cultivable microbes from midgut and hindgut contents.

| Isolates | Gene | Query length (bp) | Similarity | Bit-Score | BLAST Accession | Best match |
|---|---|---|---|---|---|---|
| CTa1L1H1 | 16S *rRNA* | 1422 | 100 | 2627.06 | JQ579628 | *Bacillus thuringiensis* |
| CTa1L1M1 | 16S *rRNA* | 1419 | 99.9 | 2617.82 | KY653110 | *Lysinibacillus fusiformis* |
| CTa1L2H2 | 16S *rRNA* | 1412 | 99.6 | 2575.35 | LC462165 | *Leminorella grimontii* |
| CTa1L2M1 | *rpoB* | 797 | 95.6 | 1282.82 | LT708255 | *Paraburkholderia caballeronis* |
| CTa1L2M2 | 16S *rRNA* | 1403 | 100 | 2591.97 | MZ676064 | *Bacillus albus* |
| CTa1L2M3 | 16S *rRNA* | 1086 | 99.5 | 1982.58 | MW802176 | *Lysinibacillus fusiformis* |
| CTa1L3M1 | 16S *rRNA* | 1406 | 92.4 | 2191.25 | EU372963 | *Bacillus* sp. |
| CZp1L1H1 | 16S *rRNA* | 1390 | 99.5 | 2540.26 | MT271951 | *Klebsiella aerogenes* |
| CZp1L1H1 | *rpoB* | 1445 | 99.6 | 2638.14 | CP024880 | *Klebsiella aerogenes* |
| CZp1L2M1 | 16S *rRNA* | 1332 | 99.2 | 2420.23 | MZ676064 | *Bacillus albus* |
| | | | | | MZ497322 | *Bacillus cereus* |
| | | | | | MN746216 | *Bacillus anthracis* |
| CZp1L2M2 | 16S *rRNA* | 825 | 99.9 | 1519.07 | OK083719 | *Bacillus cereus* |
| | | | | | OK073274 | *Bacillus paramycoides* |
| | | | | | OK056287 | *Bacillus thuringiensis* |
| CZp1L2H3 | 16S *rRNA* | 1456 | 99.5 | 2643.68 | CP077291 | *Citrobacter farmeri* |
| CZp1L3H1 | 16S *rRNA* | 1094 | 99.5 | 1999.2 | MZ914647 | *Lysinibacillus fusiformis* |
| | | | | | KY407914 | *Lysinibacillus sphaericus* |
| CZp1L3M1 | 16S *rRNA* | 1151 | 99.7 | 2113.69 | MZ934752 | *Bacillus cereus* |
| | | | | | MW857094 | *Bacillus pacificus* |
| | | | | | MW850424 | *Bacillus nitratireducens* |
| CZp1L3H3 | 16S *rRNA* | 1090 | 99.8 | 2001.04 | GU125641 | *Lysinibacillus fusiformis* |
| CZp1L4H2 | *rpoB* | 1447 | 99.5 | 2632.6 | CP077291 | *Citrobacter farmeri* |
| DLs1L1M1 | 16S *rRNA* | 1419 | 99.9 | 2617.82 | MZ497322 | *Bacillus cereus* |
| | | | | | MZ310685 | *Bacillus albus* |
| | | | | | MW931846 | *Bacillus tropicus* |
| | | | | | MW380582 | *Bacillus thringeniensis* |
| DLs1L1M2 | 16S *rRNA* | 1405 | 99.8 | 2580.89 | MG049778 | *Lysinibacillus* sp. |
| DLs1L1M3 | 16S *rRNA* | 1060 | 99.9 | 1951.18 | MT355730 | *Bacillus* sp. |
| DLs1L1H2 | 16S *rRNA* | 1396 | 99.8 | 2558.73 | KX357798 | *Citrobacter koseri* |
| DLs1L2M1 | 16S *rRNA* | 1419 | 99.9 | 2617.82 | MZ497322 | *Bacillus cereus* |
| | | | | | MZ310685 | *Bacillus albus* |
| | | | | | MW931846 | *Bacillus tropicus* |
| | | | | | MW380582 | *Bacillus thuringiensis* |

| | | | | | | |
|---|---|---|---|---|---|---|
| DLs1L2H1 | 16S *rRNA* | 1406 | 99.7 | 2577.2 | NR_112628 | *Lysinibacillus fusiformis* |
| DLs1L2H2 | 16S *rRNA* | 1405 | 99.4 | 2551.34 | NR_112628 | *Lysinibacillus fusiformis* |
| DLs1L2H3 | 16S *rRNA* | 1367 | 98.5 | 2407.3 | MF521962 | *Lysinibacillus fusiformis* |
| DLs1L3M1 | 16S *rRNA* | 1042 | 98.8 | 1862.54 | MH532492 | *Citrobacter koseri* |
| DLs1L3H2 | 16S *rRNA* | 1409 | 99.8 | 2584.58 | KY561619 | *Bacillus* sp. |
| DLs1L3H3 | 16S *rRNA* | 1027 | 99.6 | 1882.86 | AB934966 | *Bacillus aryabhattai* |
| LJf1L1M1 | *rpoB* | 1479 | 99.7 | 2710.16 | CP038262 | *Acinetobacter baumannii* |
| LJf1L1M1 | 16S *rRNA* | 1411 | 99.9 | 2603.05 | MT673788 | *Bacillus subtilis* |
| | | | | | MK713704 | *Bacillus tequilensis* |
| | | | | | MG651202 | *Bacillus halotolerans* |
| | | | | | KT986166 | *Bacillus amyloliquefaciens* |
| | | | | | KT986156 | *Bacillus axarquiensis* |
| | | | | | KT986983 | *Geobacillus stearothermophilus* |
| LJf1L1M2 | *rpoB* | 1481 | 99.7 | 2713.85 | CP038262 | *Acinetobacter baumannii* |
| LJf1L2H1 | 16S *rRNA* | 694 | 99.1 | 1243.92 | LC183868 | *Lysinibacillus macroides* |
| LJf1L2H2 | 16S *rRNA* | 1406 | 99.8 | 2580.89 | MZ734317 | *Bacillus safensis* |
| | | | | | MZ489518 | *Bacillus australimaris* |
| | | | | | MZ328872 | *Bacillus stratosphericus* |
| | | | | | MN750426 | *Bacillus pumilus* |
| LJf1L3M1 | 16S *rRNA* | 1413 | 99.7 | 2588.28 | NR_025333 | *Leminorella grimontii* |
| LJf1P1H2 | 16S *rRNA* | 1437 | 90.7 | 1912.4 | CP072199 | *Serratia marcescens* |
| LJf1P3H1 | 16S *rRNA* | 1436 | 99.4 | 2603 | CP066089 | *Citrobacter koseri* |
| QSc1L2H1P | ITS rDNA | 476 | 95.6 | 776.713 | MN904886 | *Candida xylanilytica* |
| SLa1LH1 | 16S *rRNA* | 1398 | 99.9 | 2579.04 | KF484685 | *Brevibacillus laterosporus* |
| SLa1LM2 | 16S *rRNA* | 1397 | 99.9 | 2577.2 | MZ923706 | *Bacillus cereus* |
| SLa1LM3 | 16S *rRNA* | 1351 | 97.9 | 2335.29 | KC954751 | *Telmatobacter* sp. |
| SLa1LM4 | 16S *rRNA* | 1353 | 99.9 | 2492.25 | MZ931308 | *Staphylococcus aureus* |
| SLa1LM5 | 16S *rRNA* | 1363 | 97.6 | 2338.98 | KC954751 | *Telmatobacter* sp. |
| SLa1LM7 | 16S *rRNA* | 1417 | 99.4 | 2579.04 | MG266284 | *Bacillus* sp. |
| SLa1LM8 | 16S *rRNA* | 1351 | 98.4 | 2375.91 | MH686091 | *Mesorhizobium* sp. |
| SLa1LM9 | 16S *rRNA* | 1362 | 97.9 | 2351.91 | KC954751 | *Telmatobacter* sp. |
| SLa1LH2 | 16S *rRNA* | 1418 | 100 | 2619.67 | MW559318 | *Bacillus cereus* |
| | | | | | MW405816 | *Bacillus mobilis* |
| | | | | | MW205819 | *Bacillus thuringiensis* |
| | | | | | KU898281 | *Bacillus toyonensis* |
| | | | | | KU877666 | *Bacillus oryzaecorticis* |
| SLa1LH5 | 16S *rRNA* | 1399 | 99.8 | 2567.96 | JQ724338 | *Thauera* sp. |

| | | | | | | |
|---|---|---|---|---|---|---|
| WLa1L1M2 | 16S *rRNA* | 1019 | 99.1 | 1847.77 | MZ312268 | *Paracoccus* sp. |
| WLa1L1H2 | 16S *rRNA* | 895 | 92.8 | 1297.47 | KR706347 | *Psedomonas flexibilis* |
| WLa1L2M3 | *rpoB* | 1503 | 90 | 1940.1 | CP033934 | *Chryseobacterium balustinum* |
| WLa1L2M3 | 16S *rRNA* | 1505 | 86.8 | 1762.82 | CP033934 | *Chryseobacterium balustinum* |
| WLc1L3H1 | 16S *rRNA* | 1400 | 99.2 | 2540.26 | MN421133 | *Bacillus xiamensis* |
| | | | | | MH396728 | *Bacillus safensis* |
| | | | | | KT005170 | *Bacillus altitudinis* |
| WLc1L2H1P | ITS rDNA | 524 | 99.8 | 961.378 | AB164370 | *Trichosporon siamense* |
| XPs1L1M1 | 16S *rRNA* | 1445 | 96.7 | 2409.15 | CP066089 | *Citrobacter koseri* |
| XPs1L1M2-2 | 16S *rRNA* | 1447 | 93 | 2115.53 | LS483470 | *Leminorella richardii* |
| XPs1L1M3 | 16S *rRNA* | 598 | 98.2 | 1051.86 | KP997262 | *Bacillus thuringiensis* |
| | | | | | FJ188319 | *Bacillus cereus* |
| XPs1L3M2 | 16S *rRNA* | 1417 | 98.6 | 2543.96 | CP063354 | *Serratia marcescens* |
| XYa1L1H3 | 16S *rRNA* | 1353 | 99.4 | 2470.09 | MZ947169 | *Priestia megaterium* |
| | | | | | MZ923713 | *Priestia aryabhattai* |
| | | | | | MZ923703 | *Bacillus zanthoxyli* |
| XYa1L1M1 | 16S *rRNA* | 1382 | 99.7 | 2534.72 | MH532492 | *Citrobacter koseri* |
| XYa1L3H2 | 16S *rRNA* | 1111 | 95.2 | 1729.58 | KY434003 | *Edaphobacter* sp. |
| XYa1L3M1 | 16S *rRNA* | 1086 | 99.8 | 1993.66 | MZ569686 | *Lysinibacillus macroides* |
| | | | | | MH385002 | *Lysinibacillus sphaericus* |
| | | | | | MG991566 | *Lysinibacillus boronitolerans* |
| | | | | | MF967404 | *Lysinibacillus fusiformis* |
| XYa1L3M2 | 16S *rRNA* | 1138 | 99.8 | 2091.53 | MW830387 | *Bacillus cereus* |
| | | | | | MW380523 | *Bacillus subtilis* |
| | | | | | MW065486 | *Bacillus paramycoides* |
| | | | | | MN882644 | *Bacillus albus* |
| XYa1L5M2 | 16S *rRNA* | 1350 | 99.8 | 2483.02 | MZ712064 | *Priestia aryabhattai* |
| | | | | | MZ569841 | *Priestia megaterium* |
| | | | | | MZ496951 | *Bacillus* sp. |
| XYa1L5H2 | 16S *rRNA* | 1420 | 99.9 | 2619.67 | MZ675431 | *Bacillus cereus* |
| XYa1L5H3 | 16S *rRNA* | 1363 | 97.3 | 2320.51 | KY434001 | *Edaphobacter* sp. |
| XYa1L5M3 | 16S *rRNA* | 1403 | 100 | 2591.97 | MZ676063 | *Bacillus albus* |
| | | | | | MZ497322 | *Bacillus cereus* |
| | | | | | MW931846 | *Bacillus tropicus* |
| XYa1L6H2 | 16S *rRNA* | 1403 | 99.8 | 2577.2 | MZ722994 | *Priestia megaterium* |
| | | | | | MZ026430 | *Bacillus* sp. |
| | | | | | MW803060 | *Priestia aryabhattai* |

| | | | | | | |
|---|---|---|---|---|---|---|
| XYc1L1H1 | 16S *rRNA* | 1403 | 99.8 | 2577.2 | KX344022 | *Bacillus altitudinis* |
| | | | | | KT986116 | *Bacillus pumilus* |
| | | | | | KT380676 | *Bacillus safensis* |
| XYc1L1H1P | ITS rDNA | 516 | 98.3 | 909.672 | MN904886 | *Candida xylanilytica* |
| XYc1L1H2 | 16S *rRNA* | 1238 | 99.5 | 2265 | MZ723093 | *Bacillus aerius* |
| | | | | | MZ713012 | *Bacillus altitudinis* |
| | | | | | MZ675499 | *Bacillus aerophilus* |
| XYc1L2M1 | 16S *rRNA* | 1283 | 99.2 | 2327.9 | KY652111 | *Bacillus* sp. |
| XYc1L2M2 | 16S *rRNA* | 1403 | 99.9 | 2588.28 | MZ676063 | *Bacillus albus* |
| | | | | | MZ497322 | *Bacillus cereus* |
| | | | | | MW931846 | *Bacillus tropicus* |
| XYc1L2M3 | 16S *rRNA* | 1118 | 97.1 | 1941.95 | MZ914647 | *Lysinibacillus fusiformis* |
| XYc1L3H1P | ITS rDNA | 516 | 98.1 | 905.979 | MN904886 | *Candida xylanilytica* |
| XYc1L4H1 | 16S *rRNA* | 1372 | 99.9 | 2521.8 | MN700259 | *Bacillus* sp. |
| XYc1L4H1P | ITS rDNA | 542 | 100 | 1002 | MZ363142 | *Candida tropicalis* |
| ZPa1L1H1 | 16S *rRNA* | 1392 | 99.7 | 2551.34 | KM016946 | *Citrobacter koseri* |
| ZPa1L1H2 | 16S *rRNA* | 1461 | 97.4 | 2486.71 | CP034752 | *Jinshanibacter zhutongyuii* |
| ZPa1L1H3 | 16S *rRNA* | 1002 | 98.4 | 1753.59 | MN057723 | *Pragia* sp. |
| ZPa1L2M2 | 16S *rRNA* | 921 | 98.4 | 1616.94 | GQ423394 | *Bacillus* sp. |
| ZPa1L2M3 | 16S *rRNA* | 1416 | 99.1 | 2542.11 | MN057723 | *Pragia* sp. |
| ZPa1L2H1 | 16S *rRNA* | 1393 | 98.1 | 2438.7 | MN057723 | *Pragia* sp. |
| ZPa1L2H2 | 16S *rRNA* | 1410 | 99.1 | 2529.18 | MN057723 | *Pragia* sp. |
| ZPa1L2H3 | 16S *rRNA* | 1396 | 98.1 | 2460.86 | MN057723 | *Pragia* sp. |
| ZPa1L3H1 | 16S *rRNA* | 1406 | 99.7 | 2577.2 | MW786727 | *Bacillus toyonensis* |
| | | | | | MW559318 | *Bacillus cereus* |
| | | | | | MW405816 | *Bacillus mobilis* |
| | | | | | MW287205 | *Bacillus thuringiensis* |
| ZPa1L3M1 | 16S *rRNA* | 574 | 99.8 | 1053.71 | MW175549 | *Citrobacter koseri* |
| ZPa1L4H2 | 16S *rRNA* | 1141 | 95.1 | 1751.74 | FJ463782 | *Citrobacter* sp. |
| ZPa1L4M1 | 16S *rRNA* | 1384 | 99.6 | 2525.49 | MT367744 | *Citrobacter koseri* |
| | | | | | MN749522 | *Citrobacter freundii* |
| ESs1L3H1P | ITS rDNA | 532 | 99.8 | 979.845 | FJ662410 | *Candida tropicalis* |
| ESs1L3H1 | 16S *rRNA* | 928 | 100 | 1714.81 | MN073814 | *Bacillus* sp. |
| ESs1L3H2 | 16S *rRNA* | 1086 | 99.9 | 1999.2 | MN428188 | *Lysinibacillus macrolides* |
| | | | | | MK318259 | *Lysinibacillus xylanilyticus* |
| | | | | | MF948384 | *Bacillus* sp. |
| ESs1L1H1 | 16S *rRNA* | 1063 | 100 | 1964.11 | JQ661039 | *Bacillus* sp. |

| | | | | | | |
|---|---|---|---|---|---|---|
| ESs1L3H3 | 16S *rRNA* | 1065 | 99.7 | 1947.49 | MN428188 | *Lysinibacillus macrolides* |
| | | | | | MK318259 | *Lysinibacillus xylanilyticus* |
| | | | | | MF948384 | *Bacillus* sp. |
| | | | | | KU983851 | *Lysinibacillus pakistanensis* |
| NPc3L2 | 16S *rRNA* | 975 | 99.8 | 1792.37 | MN416232 | *Bacillus toyonensis* |
| | | | | | KP813752 | *Bacillus thuringiensis* |
| | | | | | KJ685393 | *Bacillus cereus* |
| NPc3L1 | 16S *rRNA* | 1408 | 99.6 | 2564.27 | CP066089 | *Citrobacter koseri* |
| LYf1L1M1 | *rpoB* | 1430 | 99.4 | 2604.9 | CP081016 | *Pseudomonas* sp. |
| LYf1L1M1 | 16S *rRNA* | 825 | 99.3 | 1485.83 | MH091341 | *Pseudomonas mosselii* |
| LYf1L2M1 | 16S *rRNA* | 942 | 99.9 | 1735.12 | LR215149 | *Bacillus cereus* |
| LYf1L3H1P | ITS rDNA | 531 | 100 | 981.691 | NR_151809 | *Candida saraburiensis* |
| LYf1L3M2P | ITS rDNA | 531 | 100 | 981.691 | NR_151809 | *Candida saraburiensis* |
| JAf1L3H1 | 16S *rRNA* | 1085 | 99.6 | 1986.27 | MW926960 | *Bacillus tequilensis* |
| | | | | | MW888885 | *Bacillus subtilis* |
| | | | | | MW776608 | *Bacillus velezensis* |
| JAf1L2H2 | 16S *rRNA* | 1383 | 99.1 | 2507.02 | CP071070 | *Citrobacter sedlakii* |
| JAf1L2H3 | 16S *rRNA* | 1407 | 99.4 | 2547.65 | MW365215 | *Serratia marcescens* |
| JAf1L2H1 | 16S *rRNA* | 1136 | 99.6 | 2084.14 | MZ540090 | *Bacillus proteolyticus* |
| | | | | | MZ485376 | *Bacillus cereus* |
| | | | | | MZ443981 | *Bacillus toyonensis* |
| | | | | | MZ411685 | *Bacillus thuringiensis* |
| | | | | | MZ310685 | *Bacillus albus* |
| JAf1L3M1P1 | ITS rDNA | 653 | 99.7 | 1195.9 | MN268781 | *Debaryomyces* sp. |
| JAf1L2M1P | ITS rDNA | 656 | 99.7 | 1201.44 | MN268781 | *Debaryomyces* sp. |
| DOs1L1H2 | 16S *rRNA* | 1055 | 99.8 | 1941.95 | MW979588 | *Fictibacillus phosphorivorans* |
| | | | | | MW363312 | *Fictibacillus barbaricus* |
| | | | | | MT829784 | *Bacillus* sp. |
| | | | | | MT704540 | *Fictibacillus arsenicus* |
| | | | | | MG651557 | *Fictibacillus halophilus* |
| | | | | | MG651493 | *Fictibacillus nanhaiensis* |
| DOs1L1H1P | ITS rDNA | 522 | 98.1 | 917.059 | MN904886 | *Candida xylanilytica* |
| DOs1L1H2P | ITS rDNA | 548 | 95.9 | 909.672 | MK394120 | *Candida sojae* |
| DOs1L1M1 | 16S *rRNA* | 1082 | 97.3 | 1820.07 | MZ312268 | *Paracoccus* sp. |
| DOs1L1M1A | 16S *rRNA* | 1074 | 99.8 | 1973.34 | KY816740 | *Brevibacillus parabrevis* |
| DOs1L1M2A | 16S *rRNA* | 1063 | 99.8 | 1951.18 | KR077844 | *Bacillus* sp. |
| DOs1L1M1P | ITS rDNA | 522 | 98.1 | 918.905 | MN904886 | *Candida xylanilytica* |

| | | | | | | |
|---|---|---|---|---|---|---|
| DOs1L1M3 | 16S *rRNA* | 1084 | 98.6 | 1938.26 | KC178941 | *Bacillus humi* |
| DOs1L1M4 | 16S *rRNA* | 1230 | 99.2 | 2230.03 | MZ468617 | *Brucella intermedia* |
| JAs1L1H2 | 16S *rRNA* | 1068 | 99.9 | 1965.96 | MZ712998 | *Bacillus cereus* |
| | | | | | MZ675479 | *Bacillus wiedmannii* |
| | | | | | MZ569844 | *Bacillus mobilis* |
| | | | | | MZ234580 | *Bacillus thuringiensis* |
| | | | | | MW786728 | *Bacillus toyonensis* |
| JAs1L1H3 | 16S *rRNA* | 1017 | 97.8 | 1770.21 | MN410691 | *Chryseobacterium* sp. |
| JAs1L1H4 | 16S *rRNA* | 1061 | 99.5 | 1941.95 | MK880382 | *Pseudomonas guariconensis* |
| | | | | | KC634234 | *Pseudomonas plecoglossicida* |
| JAs1L1H1P | ITS rDNA | 470 | 100 | 869.046 | AB164370 | *Trichosporon siamense* |
| JAs1L1H1A | 16S *rRNA* | 1003 | 97.8 | 1762.82 | NR_044932 | *Microbacterium arabinogalactanolyticum* |
| | | | | | MF196241 | *Microbacterium esteraromaticum* |
| | | | | | KM488483 | *Microbacterium oxydans* |
| JAs1L1H2A | 16S *rRNA* | 1055 | 98.2 | 1879.16 | MH894224 | *Bacillus oceanisedimins* |
| | | | | | KT875347 | *Bacillus firmus* |
| JAs1L2H1 | *rpoB* | 1457 | 93.7 | 2180.17 | LS483470 | *Leminorella richardii* |
| JAs1L2H1 | 16S *rRNA* | 1226 | 95.9 | 2023.2 | NR_025333 | *Leminorella grimontii* |
| JAs1L2H2 | 16S *rRNA* | 1063 | 100 | 1964.11 | JN084129 | *Bacillus asahii* |
| JAs1L2H3 | 16S *rRNA* | 1171 | 99.6 | 2139.54 | MZ047974 | *Citrobacter amalonaticus* |
| JAs1L2M2 | 16S *rRNA* | 1063 | 99.9 | 1958.57 | JQ661039 | *Bacillus* sp. |
| JAs1L2M1A | 16S *rRNA* | 1061 | 99.8 | 1949.34 | JX847112 | *Lysinibacillus* sp. |
| JAs1L2M2A | 16S *rRNA* | 911 | 98.1 | 1594.78 | AB098571 | *Arthrobacter* sp. |
| JAs1L2M1P | ITS rDNA | 640 | 100 | 1182.98 | MK394112 | *Candida palmioleophila* |
| JAs1L2M2P | ITS rDNA | 524 | 99.8 | 961.378 | AB164370 | *Trichosporon siamense* |
| JAs1L2M3P | ITS rDNA | 537 | 100 | 992.771 | MZ363142 | *Candida tropicalis* |
| | | | | | KT715474 | *Candida dubliniensis* |
| JAs1L2M4P | ITS rDNA | 527 | 100 | 974.305 | NR_151809 | *Candida saraburiensis* |
| JAs1L2H1A | 16S *rRNA* | 1019 | 99.8 | 1875.47 | MZ9347532 | *Bacillus cereus* |
| | | | | | MZ569844 | *Bacillus mobilis* |
| | | | | | MZ540090 | *Bacillus proteolyticus* |
| | | | | | MZ520364 | *Bacillus pacificus* |
| JAs1L2H2A | 16S *rRNA* | 1068 | 99.6 | 1954.88 | KY816740 | *Brevibacillus parabrevis* |
| JAs1L2H4A | 16S *rRNA* | 981 | 99.7 | 1796.06 | MZ914416 | *Bacillus mycoides* |
