# Supplementary material for: Coconut rhinoceros beetle digestive symbiosis with potential plant cell wall degrading microbes
Source: NPJ Biofilms Microbiomes. 2024 Mar 30;10:34. doi: 10.1038/s41522-024-00505-9 (PMC10981690; doi:10.1038/s41522-024-00505-9)
Supplement: Supplementary file 2 — Reporting Summary [file 41522_2024_505_MOESM2_ESM.pdf]

Corresponding author(s): Chiao-Jung Han

Last updated by author(s): Feb 1, 2024

Reporting Summary

Nature Portfolio wishes to improve the reproducibility of the work that we publish. This form provides structure for consistency and transparency in reporting. For further information on Nature Portfolio policies, see our Editorial Policies and the Editorial Policy Checklist.

Statistics

For all statistical analyses, confirm that the following items are present in the figure legend, table legend, main text, or Methods section.

n/a	Confirmed	
☐	☒	The exact sample size (*n*) for each experimental group/condition, given as a discrete number and unit of measurement
☐	☒	A statement on whether measurements were taken from distinct samples or whether the same sample was measured repeatedly
☐	☒	The statistical test(s) used AND whether they are one- or two-sided<br>*Only common tests should be described solely by name; describe more complex techniques in the Methods section.*
☐	☒	A description of all covariates tested
☒	☐	A description of any assumptions or corrections, such as tests of normality and adjustment for multiple comparisons
☐	☒	A full description of the statistical parameters including central tendency (e.g. means) or other basic estimates (e.g. regression coefficient) AND variation (e.g. standard deviation) or associated estimates of uncertainty (e.g. confidence intervals)
☐	☒	For null hypothesis testing, the test statistic (e.g. *F*, *t*, *r*) with confidence intervals, effect sizes, degrees of freedom and *P* value noted<br>*Give P values as exact values whenever suitable.*
☒	☐	For Bayesian analysis, information on the choice of priors and Markov chain Monte Carlo settings
☒	☐	For hierarchical and complex designs, identification of the appropriate level for tests and full reporting of outcomes
☒	☐	Estimates of effect sizes (e.g. Cohen's *d*, Pearson's *r*), indicating how they were calculated

Our web collection on statistics for biologists contains articles on many of the points above.

Software and code

Policy information about availability of computer code

Data collection	Package dplyr, ggplot2, corrplot, rstatix, vegan, igraph, and pairwiseAdonis in R; QIIME2 and PICRUSt2
Data analysis	Package dplyr, ggplot2, corrplot, rstatix, vegan, igraph, and pairwiseAdonis in R; QIIME2 and PICRUSt2

For manuscripts utilizing custom algorithms or software that are central to the research but not yet described in published literature, software must be made available to editors and reviewers. We strongly encourage code deposition in a community repository (e.g. GitHub). See the Nature Portfolio guidelines for submitting code & software for further information.

Data

Policy information about availability of data

All manuscripts must include a data availability statement. This statement should provide the following information, where applicable:

- Accession codes, unique identifiers, or web links for publicly available datasets
- A description of any restrictions on data availability
- For clinical datasets or third party data, please ensure that the statement adheres to our policy

The datasets generated and/or analyzed during the current study are available under the BioProject PRJNA997560 in the NCBI GenBank, [https://www.ncbi.nlm.nih.gov/bioproject/PRJNA997560].

Research involving human participants, their data, or biological material

Policy information about studies with human participants or human data. See also policy information about sex, gender (identity/presentation), and sexual orientation and race, ethnicity and racism.

Reporting on sex and gender	This study did not involve any clinical trials or patients, so this is not relevant to our research.
Reporting on race, ethnicity, or other socially relevant groupings	This study did not involve any clinical trials or patients, so this is not relevant to our research.
Population characteristics	This study did not involve any clinical trials or patients, so this is not relevant to our research.
Recruitment	This study did not involve any clinical trials or patients, so this is not relevant to our research.
Ethics oversight	This study did not involve any clinical trials or patients, so this is not relevant to our research.

Note that full information on the approval of the study protocol must also be provided in the manuscript.

Field-specific reporting

Please select the one below that is the best fit for your research. If you are not sure, read the appropriate sections before making your selection.

☐ Life sciences ☐ Behavioural & social sciences ☒ Ecological, evolutionary & environmental sciences

For a reference copy of the document with all sections, see nature.com/documents/nr-reporting-summary-flat.pdf

Ecological, evolutionary & environmental sciences study design

All studies must disclose on these points even when the disclosure is negative.

Study description	Profile the microbiomes of Oryctes rhinoceros larvae around Taiwan
Research sample	Oryctes rhinoceros larvae
Sampling strategy	Choose the late instar larvae
Data collection	Chiao-Jung Han recorded the collection information, including the collecting site and host plant(habitat), and GPS.
Timing and spatial scale	Samples for profiling the microbiomes of CRB around Taiwan were collected from 7/4/2020 to 7/8/2022. Samples for the microbiomes of developmental stages were collected on 4/6/2023.
Data exclusions	No data were excluded from the analyses.
Reproducibility	For each location, we sampled at least three individuals as possible. For the microbiomes of developmental stages, we used three Female adults, and nine of the eggs, early instar larvae, 2nd instar and 3rd instar larvae, respectively.
Randomization	We extracted all collected samples, so no randomization problem is expected to occur.
Blinding	This study did not involve any clinical trials or patients, so the blinding procedures may not be relevant to our research.

Did the study involve field work? ☒ Yes ☐ No

Field work, collection and transport

Field conditions	Temperature in field usually at 25-35 degrees of Celsius.
Location	All samples are collected in Taiwan.
Access & import/export	Every sample is collected from the field with the agreement of the field owner.
Disturbance	The rotten wood, where the beetle likely hides, will be chopped into pieces, and we need to clean up the field after each collection.

Reporting for specific materials, systems and methods

We require information from authors about some types of materials, experimental systems and methods used in many studies. Here, indicate whether each material, system or method listed is relevant to your study. If you are not sure if a list item applies to your research, read the appropriate section before selecting a response.

Materials & experimental systems

n/a	Involved in the study	
☐	☒	Antibodies
☐	☒	Eukaryotic cell lines
☒	☐	Palaeontology and archaeology
☐	☒	Animals and other organisms
☒	☐	Clinical data
☒	☐	Dual use research of concern
☒	☐	Plants

Methods

n/a	Involved in the study	
☒	☐	ChIP-seq
☒	☐	Flow cytometry
☒	☐	MRI-based neuroimaging

Antibodies

Antibodies used	anti-V5-HRP antibody (Invitrogen)
Validation	The Anti-V5 Antibody allows detection of recombinant proteins containing the V5 epitope. This epitope is found in the P and V proteins of the paramyxovirus, SV5 (Southern et al., 1991). The Anti-V5 Antibody recognizes the 14 amino acid sequence: Gly-Lys-Pro-Ile-Pro-Asn-Pro-Leu-Leu-Gly-Leu-Asp-Ser-Thr.<br>Detailed protocols for using the antibody are in the technical manual available or in published references (Ausubel et al., 1994; Harlow & Lane, 1988).<br>The Anti-V5 Antibody (R960-25) is a mouse monoclonal IgG2a antibody. Anti-V5-HRP Antibody (R961-25) is prepared by crosslinking the Anti-V5 Antibody with horseradish peroxidase (HRP) using glutaraldehyde.<br>Sufficient antibody (50 µl) is supplied to perform 25 western immunodetections (assuming 10 ml buffer per blot).

Eukaryotic cell lines

Policy information about cell lines and Sex and Gender in Research

Cell line source(s)	sf9 cell line (Invitrogen) from the pupal ovarian tissue of the fall army worm, Spodoptera frugiperda.
Authentication	The cell lines were maintained in a sterile cell line lab for cultivation, and a daily check for contamination was conducted using a microscope.
Mycoplasma contamination	The cell line were not tested for Mycoplasma contamination
Commonly misidentified lines<br>(See ICLAC register)	NA

Animals and other research organisms

Policy information about studies involving animals; ARRIVE guidelines recommended for reporting animal research, and Sex and Gender in Research

Laboratory animals	Oryctes rhinoceros
Wild animals	Oryctes rhinoceros, caught with bug catching net and transported in plastic container at 25-30 degrees of Celsius.
Reporting on sex	Since the majority of our collection consists of larvae, it is challenging to differentiate their sex. Therefore, this information is not applicable.
Field-collected samples	The samples were incubated in a plastic container, one container only contains one individual. They were incubated at 27-30 degrees of Celsius after collection.
Ethics oversight	No ethical approval or guidance is required, as our study did not involve any clinical trials, patients, or protected species.

Note that full information on the approval of the study protocol must also be provided in the manuscript.

Plants

Seed stocks	*Report on the source of all seed stocks or other plant material used. If applicable, state the seed stock centre and catalogue number. If plant specimens were collected from the field, describe the collection location, date and sampling procedures.*
Novel plant genotypes	*Describe the methods by which all novel plant genotypes were produced. This includes those generated by transgenic approaches, gene editing, chemical/radiation-based mutagenesis and hybridization. For transgenic lines, describe the transformation method, the number of independent lines analyzed and the generation upon which experiments were performed. For gene-edited lines, describe the editor used, the endogenous sequence targeted for editing, the targeting guide RNA sequence (if applicable) and how the editor was applied.*
Authentication	*Describe any authentication procedures for each seed stock used or novel genotype generated. Describe any experiments used to assess the effect of a mutation and, where applicable, how potential secondary effects (e.g. second site T-DNA insertions, mosiacism, off-target gene editing) were examined.*
